# Endogenous green technology innovation and diffusion with strategic international spillovers

**DOI:** 10.1186/s13021-025-00376-3

**Published:** 2025-12-12

**Authors:** Zhuo Feng

**Affiliations:** School of Economics, University of Chinese Academy of Social Sciences, No. 11 Changyu Street, Fangshan District, Beijing, China

**Keywords:** Endogenous growth, Directed technical change, International spillovers, Environmental policy, Network analysis

## Abstract

Escalating environmental challenges necessitate accelerated green technology innovation and diffusion. This paper introduces a novel theoretical framework synthesizing differential game theory, endogenous growth with directed technical change, and network analysis to investigate the interplay between strategic national R&D investments, the endogenous direction of innovation, and structured international knowledge spillovers. The model contrasts non-cooperative and cooperative equilibria, revealing that non-cooperation yields suboptimal global outcomes: underinvestment in green R&D, delayed transitions, and a failure to curb long-term pollution, driven by free-riding on environmental benefits and knowledge spillovers. The spillover network’s architecture critically mediates these dynamics. Conversely, cooperative solutions markedly improve environmental and technological trajectories. Numerical simulations confirm these findings and demonstrate that globally coordinated policy mixes, specifically carbon pricing combined with green R&D subsidies, can effectively approach cooperative outcomes. The analysis underscores the critical roles of relative green R&D productivity and spillover intensity in determining the pace and success of the global green transition. This research provides a comprehensive lens for understanding and shaping the global green technology landscape and formulating effective international environmental and technology policies.

## Introduction

The escalating challenge of climate change necessitates a profound transformation of the global economy’s technological base, driven by green technology innovation and diffusion. This transition is not merely a technical challenge but represents one of the most significant economic realignments of the twenty-first century, demanding unprecedented capital investment and policy coordination. A substantial body of economic research has established that achieving this transformation efficiently requires harnessing market forces through policy, particularly by steering innovation toward environmentally sustainable technologies. It is at the intersection of international policy, endogenous innovation, and technology diffusion that the greatest challenges and opportunities lie. While this complex transformation involves myriad factors, this paper argues that its trajectory is critically shaped by three interacting core economic mechanisms: strategic international competition [17], the endogenous direction of innovation toward cleaner technologies [4], and the complex pathways of international knowledge spillovers [23]. We select these three pillars as they represent the distinct but interdependent dynamics at the global (strategic interaction), national (innovation direction), and transnational (diffusion network) levels, respectively. While critical, these mechanisms are typically studied in isolation, obscuring the feedback loops and strategic dynamics that arise from their interplay. This emphasis does not deny other drivers; rather, it isolates the interplay that is both theoretically identifiable in a dynamic game and quantitatively salient for international policy design. In our simulations, other drivers operate through these three mechanisms, allowing us to map policy levers to transition outcomes without over-extending the state space.

Policymakers and firms have intensified efforts to accelerate green innovation and diffusion as climate risks and transition commitments mount. A large body of empirical and quantitative research shows that policy instruments—both demand-pull (e.g., carbon pricing and standards) and supply-push (e.g., R&D subsidies)—reallocate inventive effort toward cleaner technologies and influence cross-border diffusion pathways. These forces interact with market size, expectations and absorptive capacity, making the transition a dynamic, multi-country problem rather than a purely domestic one [4, 7, 20, 29]. Theoretical developments mirror this practical complexity. Dynamic environmental games emphasize strategic interdependence and the time profile of policies [22, 26]. Endogenous-growth models with directed technical change explain how prices and market size govern the direction of R&D between “clean” and “dirty” technologies [5]. Research on international technology diffusion has moved from a global-pool view to network-based spillovers operating along trade, FDI, and knowledge links [11, 14]. Our contribution is to integrate these strands into a single dynamic framework to study how strategic R&D choices, the direction of innovation, and network architecture jointly shape global transition dynamics.

Existing literature reflects this disciplinary fragmentation. The rich tradition of using differential games to model international environmental agreements often treats technology as an exogenous parameter or a simple accumulating stock, thus overlooking the directed nature of innovation [17]. Conversely, models of directed technical change (DTC), while providing deep insights into the price and market size effects that steer R&D, typically operate within a single-economy context, abstracting from the strategic international R&D competition [4]. Finally, studies on technology spillovers have increasingly recognized the importance of network structures but have seldom integrated these insights into dynamic, game-theoretic models of environmental policy [14, 23].

This paper bridges these gaps by developing and analyzing a unified theoretical framework that synthesizes these three pillars. We ask: How do strategic R&D investment decisions, the endogenous direction of innovation, and the architecture of international spillover networks jointly determine the trajectory of global green technology development? Our primary contribution lies in the novel integration of these frameworks, which allows for a richer analysis of strategic behaviors, such as free-riding on networked knowledge, that are otherwise missed.

To explore this question, we construct a dynamic model of N countries engaging in a differential game. Each country strategically allocates R&D investment between "green" and "brown" technologies, driving endogenous technical change. The resulting knowledge accumulates domestically and diffuses internationally through a structured spillover network, affecting the R&D productivity of other nations. We characterize the non-cooperative (Nash) and cooperative equilibria and, recognizing the model’s analytical complexity, employ numerical simulations to explore the system’s transitional dynamics and evaluate policy interventions.

The paper proceeds as follows. "Literature review" section reviews the relevant literature. "The model" section develops the model, which is then analyzed in "Model analysis and solutions" section. "Numerical simulation framework" section presents the numerical simulation framework and results, "Policy implications" section discusses policy implications, and "Conclusion" section concludes.

## Literature review

This research builds upon and seeks to integrate three distinct but related strands of economic literature: (1) differential game theory applied to international environmental problems; (2) endogenous growth theory, particularly models of directed technical change in response to environmental concerns; and (3) the study of international technology spillovers, with an increasing focus on network analysis.

### Differential game theory in international environmental problems

Differential game theory is the natural framework for analyzing strategic environmental interactions over time, with solution concepts ranging from Open-Loop to Markov Perfect Nash Equilibria [17, 22]. While these models offer powerful insights into pollution control games and coalition formation (e.g., [10, 26]), a persistent limitation is their treatment of technology. Technological progress is often assumed to be exogenous or accumulates as a simple stock, failing to capture the crucial endogenous direction of innovation, which is a central focus of our work. The literature on R&D competition using differential games also provides insights into strategic investment behavior, though often without explicit environmental considerations.

More recent applications have refined these models to include stochastic shocks, asymmetric information, and the formation of climate coalitions (e.g., [10]). However, a persistent limitation, even in these advanced models, is the treatment of technology as either an exogenous parameter or a simple stock that accumulates without considering the endogenous direction of innovation, a gap our framework aims to fill.

### Endogenous growth, directed technical change, and the environment

The theory of endogenous growth, pioneered by Romer [30] and Aghion and Howitt [5], revolutionized economic thinking by modeling technological progress as an outcome of deliberate R&D activities rather than an exogenous process. This framework is crucial for understanding long-run economic responses to environmental challenges, as it allows for the analysis of how policies can foster green innovation.

A particularly relevant strand of this literature is the theory of directed technical change (DTC), most notably developed by Acemoglu [1] and applied to environmental economics by Acemoglu et al. [4]. These models typically feature two types of technologies: "clean" (environmentally friendly) and "dirty" (polluting). Firms or research labs decide how to allocate R&D resources between improving these two types of technologies. This allocation is driven by two main forces: the price effect (innovating in technologies for more expensive inputs is more profitable) and the market size effect (innovating for inputs with a larger market is more profitable). The elasticity of substitution between clean and dirty inputs plays a critical role in determining which effect dominates and, consequently, the direction of innovation. Environmental policies, such as carbon taxes or R&D subsidies, can alter these relative profit abilities and redirect technical change towards cleaner alternatives. Other R&D-based growth models have also incorporated environmental quality and pollution, exploring how environmental policies affect growth and welfare, often through mechanisms like labor reallocation between production and R&D sectors (e.g., [29]). Some models also consider the distinction between quantity-oriented and quality-oriented innovation, where the latter may be less environmentally damaging [5].

The DTC framework has been extended recently to explore issues such as the role of automation [2] and the interplay between innovation and firm-level markups [16]. Within the environmental sphere, a robust body of empirical evidence confirms that both carbon pricing and subsidies effectively redirect innovation [6], with recent work further highlighting the powerful role of policy expectations and market-based instruments in steering R&D towards green technologies [3]. Our model builds upon this rich literature by embedding the DTC mechanism within a multi-country strategic game, thereby endogenizing policy itself, or at least the response to it, on a global scale.

### International technology spillovers and network analysis

The global diffusion of technology, critical for the green transition, often occurs through international spillovers. While early models conceptualized these as flowing from a uniform global knowledge pool (e.g., [14]), a more recent consensus recognizes that knowledge diffuses through a complex, structured international R&D spillover network In this paradigm, countries are nodes, and channels such as trade or foreign direct investment form links whose strength and direction shape diffusion patterns. The architecture of this network—for instance, the centrality of certain countries—can fundamentally alter technological trajectories, with the effectiveness of spillovers also depending on the recipient nation’s absorptive capacity [15, 23].

Empirically, this network perspective has matured significantly. Recent studies use granular data from patent citations and trade flows to map these networks, revealing non-random, complex topologies such as "core-periphery" structures and community clustering [8], with new methodologies emerging to better identify the diffusion pathways of specific green technologies [9]. Yet, a critical gap persists: these empirically-grounded insights into network architecture are seldom integrated into the dynamic, game-theoretic models used to analyze international environmental policy. Our paper addresses this nexus by explicitly embedding a structured spillover network within a strategic game of directed technical change, allowing the network’s properties to mediate R&D incentives and free-riding behavior.

Our model builds on this by systematically analyzing how these empirically-observed network architectures mediate the strategic incentives in international environmental agreements. This aligns with a growing body of recent work that leverages network structures to understand economic dynamics, for instance, in the context of production networks and shock propagation [13] or the diffusion of climate policies [12]. Our contribution is to embed such a network structure within a dynamic game of endogenous green innovation.

### Positioning the current paper

Our research is positioned at the confluence of these three literature strands. It addresses the limitations of prior work by integrating the strategic interactions of differential games, the innovation dynamics of DTC models, and the structured diffusion pathways of network analysis into a single, dynamic framework. The preceding review highlights a clear fragmentation in the literature. The strategic interactions modeled in differential games lack endogenous and directed technical change. The rich innovation dynamics from DTC models typically unfold in a non-strategic, single-economy setting. Finally, while network analysis provides the tools to map complex spillover structures, these insights are seldom integrated into dynamic games of international R&D policy. Our paper contributes by building a unified framework at this very nexus, allowing us to analyze how strategic behavior, directed innovation, and network architecture co-determine the fate of the global green transition.

## The model

**Fig. 1:**
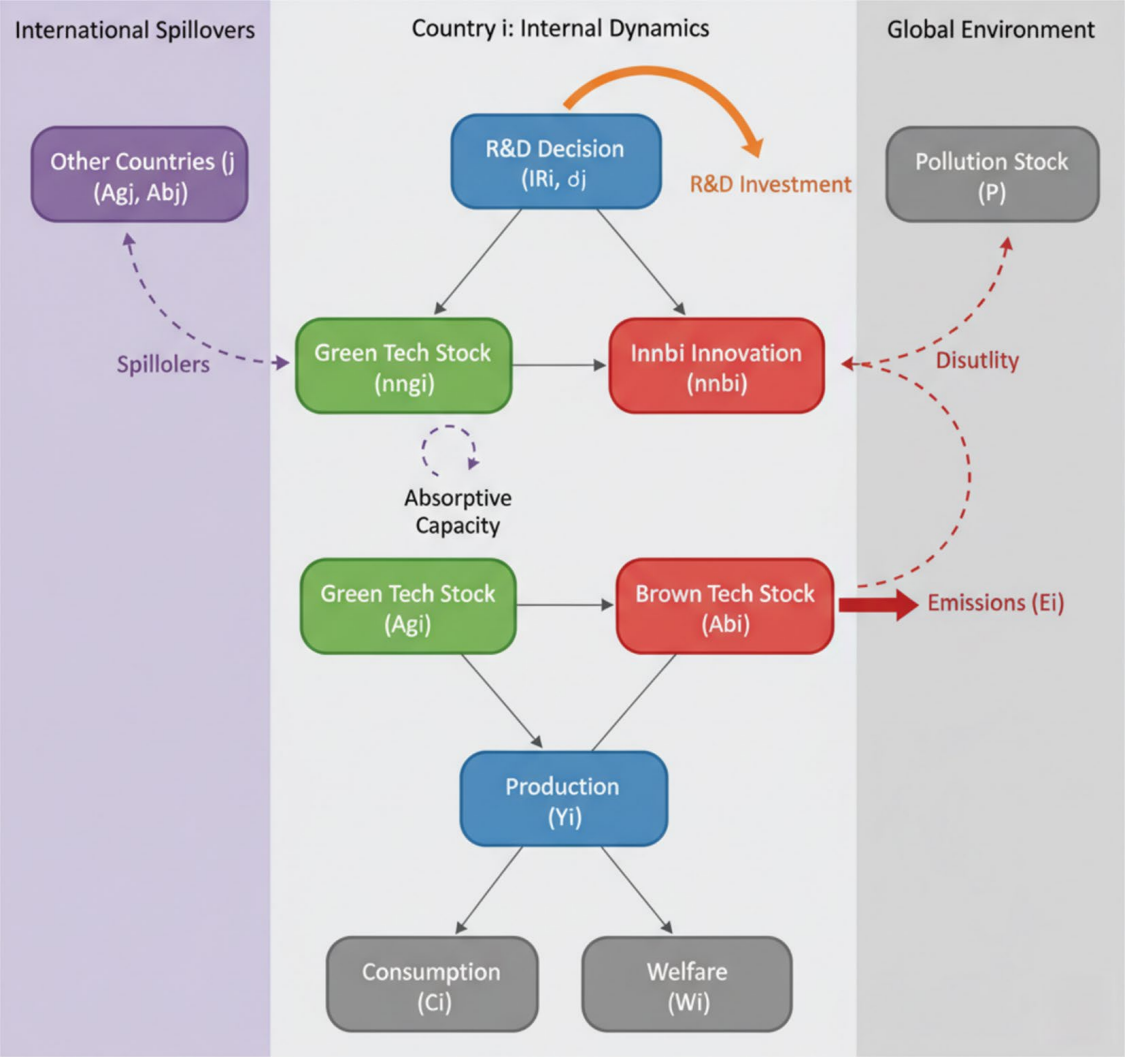
Schematic diagram of the model structure

This section develops a theoretical model of endogenous green technology innovation and diffusion with strategic international spillovers. To enhance clarity and provide an intuitive overview of the model’s architecture, Fig. 1 presents a schematic diagram illustrating the key relationships and feedback loops within and between countries.

### The economic environment

We consider a world economy with $N\ge 2$ countries, indexed by $i=1,\dots ,N$, interacting over continuous time $t$ in:

1 $${u}_{i}({C}_{i}(t),P(t))=ln{C}_{i}(t)-\frac{\phi }{2}P(t{)}^{2}$$

where $\phi>0$ is a parameter measuring the marginal disutility of pollution. The objective of country $i$ is to maximize:

2 $${W}_{i}={\int }_{0}^{\infty } {e}^{-{\rho }_{i}t}{u}_{i}\left({C}_{i}\left(t\right),P\left(t\right)\right)dt$$

*Production*: Each country $i$ produces a single final good ${Y}_{i}\left(t\right)$ using a combination of "green" intermediate inputs ${Y}_{gi}\left(t\right)$ and "brown" intermediate inputs ${Y}_{bi}\left(t\right)$. The production function is of the Constant Elasticity of Substitution (CES) type, following Acemoglu et al. [4]:

3 $${Y}_{i}\left(t\right)={\left[{\alpha }_{Y}{Y}_{gi}(t{)}^{\frac{\epsilon -1}{\epsilon }}+(1-{\alpha }_{Y}){Y}_{bi}(t{)}^{\frac{\epsilon -1}{\epsilon }}\right]}^{\frac{\epsilon }{\epsilon -1}}$$

where ${\alpha }_{Y}\in \left(\mathrm{0,1}\right)$ is the distribution parameter, and $\epsilon>0$ is the elasticity of substitution between green and brown inputs. The value of $\epsilon$ is critical: if $\epsilon>1$, green and brown inputs are substitutes; if $\epsilon <1$, they are complements, in which case phasing out brown inputs is far more difficult and costly, as they are essential to the production process alongside green inputs. As highlighted by Acemoglu et al. [4], in the case of complementarity, much stronger policy intervention is required to achieve a green transition, representing a critical boundary condition for our policy conclusions. Our simulations focus on the more optimistic case of $\epsilon>1$.

Production of green and brown inputs depends on the respective technology stocks ${A}_{gi}\left(t\right)$ and ${A}_{bi}\left(t\right)$ and labor (or a composite resource) ${L}_{gi}\left(t\right)$ and ${L}_{bi}\left(t\right)$ allocated to their production:

4 $${Y}_{gi}\left(t\right)={A}_{gi}\left(t\right){L}_{gi}\left(t\right)$$

5 $${Y}_{bi}\left(t\right)={A}_{bi}\left(t\right){L}_{bi}\left(t\right)$$

Each country has a fixed total labor supply ${L}_{i}$. For analytical tractability, our main model assumes a fixed allocation of this labor between production, ${L}_{Pi}(t),$ and R&D, ${L}_{Ri}(t)$, such that ${L}_{i}={L}_{Pi}+{L}_{Ri}$. Within production, labor is mobile between the green and brown sectors, so ${L}_{gi}(t)+{L}_{bi}(t)={L}_{Pi}(t).$

We recognize this as a significant simplification for analytical tractability. Relaxing this assumption would introduce a critical dynamic trade-off, endogenizing the allocation of labor between production and innovation. In such a setting, the relative returns to production versus R&D would govern the labor split. For example, a successful green innovation could raise the marginal product of labor in the green production sector, drawing labor away from R&D and potentially slowing future innovation—a crowding-out effect. Conversely, higher anticipated returns from future innovations, spurred by policy, could pull labor into R&D. This would create a complex general equilibrium feedback loop influencing the speed of the green transition. While our current simplification allows us to focus squarely on the strategic direction of a given R&D budget, which is the core of our inquiry, analyzing the endogenous determination of the level of the R&D labor force is a valuable direction for future research.

Consumption and R&D Investment: The final good ${Y}_{i}\left(t\right)$ can be used for consumption ${C}_{i}\left(t\right)$ or invested in R&D, ${I}_{Ri}\left(t\right)$:

6 $${Y}_{i}\left(t\right)={C}_{i}\left(t\right)+{I}_{Ri}\left(t\right)$$

The cost of R&D investment is assumed to be quadratic, representing convex adjustment costs or diminishing returns to the scale of R&D effort at a point in time:

7 $$Cost({I}_{Ri}(t))=\frac{{\chi }_{i}}{2}{I}_{Ri}(t{)}^{2}$$

where $${\chi }_{i}>0$$.

*Pollution*: Production using brown inputs generates emissions Ei (t) proportional to its scale:

8 $${E}_{i}\left(t\right)=\xi {Y}_{bi}\left(t\right)$$

where $\xi>0$ is the emission intensity of brown production. Green input production is assumed to be emission-free. The global pollution stock $P\left(t\right)$ accumulates according to:

9 $$\dot{P}\left(t\right)=\sum_{j=1}^{N} {E}_{j}\left(t\right)-{\delta }_{P}P\left(t\right)$$

where ${\delta }_{P}>0$ is the natural decay rate of the pollution stock. $P(0)={P}_{0}$ is given.

This includes state variables (${A}_{gi}(t),{A}_{bi}(t),P(t)$), control variables (${I}_{Ri}(t),{\theta }_{i}(t)$), policy variables (e.g., carbon tax ${\tau }_{i}(t)$, R&D subsidies ${s}_{gi}(t),{s}_{bi}(t)$ if modeled as controls), and parameters (e.g., ${\rho }_{i},\phi ,{\chi }_{i},{\alpha }_{Y},\epsilon ,\xi ,{\delta }_{P},$ R&D productivity parameters ${\eta }_{gi},{\eta }_{bi}$, knowledge accumulation parameters ${\nu }_{1},{\nu }_{2},{\delta }_{A},$ spillover coefficients ${\omega }_{g,ij},{\omega }_{b,ij},$ and value functions).

### Core model A: differential game of green R&D and policy

The strategic interaction between countries forms the core of the differential game.

*State Variables*: For each country $i$, the state variables are its stock of green knowledge ${A}_{gi}(t)$ and brown knowledge ${A}_{bi}(t)$. The common state variable for all countries is the global pollution stock $P(t)$. The overall state vector for the game is (${\mathbf{A}}_{\mathbf{g}}(t),{\mathbf{A}}_{\mathbf{b}}(t),P(t)$), where ${\mathbf{A}}_{\mathbf{g}}=({A}_{g1},\dots ,{A}_{gN})$ and ${\mathbf{A}}_{\mathbf{b}}=({A}_{b1},\dots ,{A}_{bN})$.

*Control Variables*: Each country $i$ chooses its total R&D investment expenditure ${I}_{Ri}(t)$ and the share of this investment allocated to green technology R&D. Thus, green R&D investment is ${\theta }_{i}(t){I}_{Ri}(t)$ and brown R&D investment is $(1-{\theta }_{i}(t)){I}_{Ri}(t)$. This variable ${\theta }_{i}(t)$ is the key control for directing technical change. For policy analysis, governments might also control instruments like a carbon tax ${\tau }_{i}(t)$ on emissions ${E}_{i}(t)$ or subsidies ${s}_{gi}(t)$ and ${s}_{bi}(t)$ to green and brown R&D respectively. Initially, we will treat these policies as parameters and later discuss scenarios where they are chosen strategically.

*Objective Function for Country* *i*: As defined in Eq. (2), country i maximizes its discounted lifetime utility, which depends on its consumption path ${C}_{i}\left(t\right)$, the global pollution path $P(t)$, and the cost of its R&D efforts. Consumption is determined by ${C}_{i}(t)={Y}_{i}(t)- {I}_{Ri}(t)$, where ${Y}_{i}(t)$ depends on ${A}_{gi}(t)$ and ${A}_{bi}(t)$ via Eqs. (3)–(5).

The equations of motion for ${A}_{gi}(t)$, ${A}_{bi}(t)$, and $P(t)$ are detailed below, incorporating endogenous growth, directed technical change, and international spillovers.

### Core model B: endogenous growth and directed technical change

Following the endogenous growth literature, technological progress results from intentional R&D investment.

*Knowledge Production Functions (Domestic Innovation)*: The flow of new domestic innovations in the green sector for country $i$, ${\mathrm{Inn}}_{gi}(t),$ depends on the R&D effort directed towards it and the existing stock of green knowledge in country $i$. A similar function applies to brown innovation.

10 $${\mathrm{Inn}}_{gi}(t)={\eta }_{gi}({\theta }_{i}(t){I}_{Ri}(t){)}^{{\nu }_{1}}({A}_{gi}(t){)}^{{\nu }_{2}}$$

11 $${Inn}_{bi}(t)={\eta }_{bi}((1-{\theta }_{i}(t)){I}_{Ri}(t){)}^{{\nu }_{1}}({A}_{bi}(t){)}^{{\nu }_{2}}$$

Here, ${\eta }_{gi}$ and ${\eta }_{bi}$ are productivity parameters for green and brown R&D, respectively. ${\nu }_{1}\in \left(\mathrm{0,1}\right]$ captures potential decreasing returns to current R&D expenditure flow. ${\nu }_{2}$ captures the intertemporal spillover from past domestic knowledge to current R&D productivity. If ${\nu }_{2}=1$, it reflects a "standing on the shoulders of giants" effect linear in the knowledge stock, akin to AK-style growth in ideas. If ${\nu }_{2}<1$, it can represent a "fishing out" effect where further innovations become harder as a technology matures. If ${\nu }_{2}>1$, it indicates accelerating returns to knowledge. For tractability, ${\nu }_{1}=1$ is often assumed. The choice of ${\theta }_{i}(t)$ determines the direction of technical change, influenced by the relative productivities (${\eta }_{gi}$,${\eta }_{bi}$) and the perceived future returns from green versus brown technologies, which depend on their current stocks ($({A}_{gi}$,${A}_{bi}$) and policy incentives.

### Core model C: networked international technology spillovers

Knowledge generated in one country can spill over to others, enhancing their technological capabilities. We model these spillovers through a network structure.

*Modeling Spillovers*: The accumulation of green knowledge in country $i$ is driven by its domestic innovation and by spillovers received from other countries $j$. The effectiveness of spillovers from country $j$ to country $i$ depends on the stock of green knowledge in country $j$, ${A}_{gj}(t)$, and a spillover coefficient ${\omega }_{g,ji}\ge 0$ (note the subscript order: from $j$ to $i$). These coefficients form an $N\times N$ green spillover matrix Ω_g_ = [ω_g,ji_]. Similarly, a matrix ${\Omega }_{g}=[{\omega }_{g,ji}]$ can define spillovers for brown technology.

The equation of motion for country $i$’s green knowledge stock, incorporating domestic innovation and international spillovers, is:

12 $$\begin{aligned} {\dot{A}}_{gi}(t) & ={\mathrm{Inn}}_{gi}(t)+\sum_{j\ne i} {\omega }_{g,ji}({A}_{gj}(t){)}^{{\zeta }_{1}}({A}_{gi}(t){)}^{{\zeta }_{2}} \\ & \quad -{\delta }_{A}{A}_{gi}(t)\end{aligned}$$

And for brown knowledge:

13 $$\begin{aligned} {\dot{A}}_{bi}(t) &={\mathrm{Inn}}_{bi}(t)+\sum_{i\ne i} {\omega }_{b,ji}({A}_{bj}(t){)}^{{\zeta }_{1}}({A}_{bi}(t){)}^{{\zeta }_{2}}\\ & \quad -{\delta }_{A}{A}_{bi}(t) \end{aligned}$$

where ${\delta }_{A}\ge 0$ is the rate of knowledge depreciation or obsolescence. The term $({A}_{gi}(t){)}^{{\zeta }_{2}}$​ in the spillover function can represent country $i$’s absorptive capacity: spillovers are more effective if country i has a higher own stock of knowledge in that domain. If ${\zeta }_{2}=0$, spillovers are independent of the recipient’s knowledge level. ${\zeta }_{1}$ captures the elasticity of spillovers with respect to the source country’s knowledge. A common simplification is ${\upzeta }_{1}=1,{\upzeta }_{2}=0$. The formulation by Liu and Ma [25], where the productivity factor of R&D itself is enhanced by a weighted sum of domestic and foreign knowledge stocks, is an alternative way to integrate spillovers directly into Eqs. (10) and (11). For example, ηgi in Eq. (10) could be replaced by ${\widetilde{\eta }}_{gi}={\eta }_{gi}^{0}(\sum_{j=1}^{N} {\omega }_{g,ji}{A}_{gj}(t){)}^{{\phi }_{S}}$, where ${\omega }_{g,ii}$ represents the effect of domestic knowledge. This formulation implies that international spillovers directly augment R&D productivity.

*Network Structure*: The matrices ${\Omega }_{g}$ and ${\Omega }_{b}$ define the international R&D spillover networks. These are taken as exogenous in the baseline model but can be based on empirical proxies such as trade intensity, geographical proximity, technological similarity, patent citation flows, or existing R&D collaboration agreements. The properties of these networks (e.g., density, centrality of certain countries, presence of clusters) are expected to significantly influence equilibrium outcomes. The properties of these networks are central to our analysis. We will characterize them using standard metrics from network science. Network Density measures the proportion of existing links to all possible links and indicates the overall level of connectivity. Node Centrality (e.g., degree centrality or eigenvector centrality) identifies countries that are major sources or recipients of spillovers. The presence of Clustering or community structures can reveal subgroups of countries with particularly strong R&D ties. These structural properties are hypothesized to significantly influence equilibrium R&D investment patterns, free-riding incentives, and the overall speed of the green transition.

*Absorptive Capacity*: As mentioned, the recipient’s own knowledge stock ${A}_{gi}$ (via ${\zeta }_{2}$) can proxy for absorptive capacity. Alternatively, absorptive capacity could be modeled as a function of human capital or specific investments in learning, though this adds further complexity.

The interaction between a country’s choice of R&D direction (${\theta }_{i}$) and the structure of the spillover networks (${\Omega }_{g}$,${\Omega }_{b}$) introduces complex strategic considerations. A nation might direct its R&D not only based on domestic priorities but also to maximize beneficial incoming spillovers or to strategically position itself within the global innovation landscape, for instance, by specializing in technologies where it can become a key knowledge exporter to partners if the corresponding spillover coefficient ${\omega }_{ij}$ is high.

This model structure, with multiple state variables per country (${A}_{gi}(t),{A}_{bi}(t)$) plus a global pollution stock ($P$), in an N-country differential game setting where R&D allocation is endogenous and spillovers are networked, presents a rich but analytically challenging framework. The high dimensionality ($2N+1$ state variables) and inherent non-linearities in innovation and environmental systems suggest that obtaining fully analytical solutions will be difficult, particularly for Markov Perfect Nash Equilibria. This motivates the subsequent exploration of numerical methods. The model is designed to analyze whether international spillovers ultimately serve as a “blessing” by accelerating the global green transition through faster diffusion, or a "curse" by encouraging free-riding on R&D and potentially perpetuating brown technologies if those also spill over effectively. The relative strengths and structures of ${\Omega }_{g}$ and ${\Omega }_{b}$ will be crucial determinants. It is crucial to note that the structures of ${\Omega }_{g}$ and ${\Omega }_{b}$ are likely asymmetric in reality. The networks for diffusing mature, carbon-intensive industrial technologies may be older, denser, and more entrenched than the emerging networks for novel green technologies. This potential asymmetry is a key factor in generating technological path dependence and “lock-in”.

## Model analysis and solutions

This section focuses on characterizing the equilibrium outcomes of the differential game defined in "The model" section. We will analyze both non-cooperative equilibria and the cooperative (socially optimal) solution.

### Non-cooperative (Nash) equilibrium

In a non-cooperative setting, each country $i$ chooses its R&D investment ${I}_{Ri}(t)$ and its allocation to green R&D ${\theta }_{i}(t)$ to maximize its own intertemporal welfare ${W}_{i}$ (Eq. 2), taking the strategies of all other countries $j\ne i$ as given.

#### General approach

The problem for each country $i$ is to:

$$\underset{{I}_{Ri}\left(t\right),{\theta }_{i}\left(t\right)}{\mathrm{max}}{\int }_{0}^{\infty }{e}^{-{\rho }_{i}t} {u}_{i}\left({C}_{i}\left(t\right),P\left(t\right)\right)dt$$

Subject to:

$${C}_{i}\left(t\right)={Y}_{i}\left({A}_{gi}\left(t\right),{A}_{bi}\left(t\right),{L}_{Pi}\left(t\right)\right)-{I}_{Ri}\left(t\right)$$

$$\dot{P}(t)=\sum_{j=1}^{N} \xi {Y}_{bj}({A}_{bj}(t),{L}_{Pbj}(t))-{\delta }_{P}P(t)$$

$$\begin{aligned} {\dot{A}}_{gi}(t) & ={\eta }_{gi}({\theta }_{i}(t){I}_{Ri}(t){)}^{{\nu }_{1}}({A}_{gi}(t){)}^{{\nu }_{2}}\\&\quad +\sum_{j\ne i} {\omega }_{g,ji}({A}_{gj}(t){)}^{{\zeta }_{1}}({A}_{gi}(t){)}^{{\zeta }_{2}}-{\delta }_{A}{A}_{gi}(t) \end{aligned}$$

$$\begin{aligned} {\dot{A}}_{bi}(t) &={\eta }_{bi}((1-{\theta }_{i}(t)){I}_{Ri}(t){)}^{{\nu }_{1}}({A}_{bi}(t){)}^{{\nu }_{2}}\\ & \quad +\sum_{j\ne i} {\omega }_{b,ji}({A}_{bj}(t){)}^{{\zeta }_{1}}({A}_{bi}(t){)}^{{\zeta }_{2}}-{\delta }_{A}{A}_{bi}(t) \end{aligned}$$

and initial conditions ${A}_{gi}(0),{A}_{bi}(0),P(0)$.

#### Open-loop Nash equilibrium (OLNE)

In an OLNE, strategies are functions of time only, ${u}_{i}^{*}(t)=({I}_{Ri}^{*}(t),{\theta }_{i}^{*}(t))$. Each country commits to these paths at $t=0$.

To derive the OLNE, we set up the current-value Hamiltonian for country $i$. Let ${\lambda }_{Pi}(t)$ be the co-state variable associated with the global pollution stock $P(t)$, ${\mu }_{gi}(t)$ be the co-state for country $i$’s green technology stock ${A}_{gi}(t)$, and ${\mu }_{bi}(t)$ for its brown technology stock ${A}_{bi}(t)$, from the perspective of country $i$.

The current-value Hamiltonian for country $i$ is:

14 $$\begin{aligned} H_{i} & = \ln \left( {C_{i} (t)} \right) - \frac{\phi }{2}P(t)^{2} - \frac{\chi_{i}}{2}I_{Ri} (t)^{2}\\ & \quad + \lambda_{Pi} (t)\left[ \mathop \sum \limits_{j = 1}^{N} \xi Y_{bj} \left( {A_{bj} (t),L_{Pbj} (t)} \right) - \delta_{P} P(t) \right] \\ & \quad + \mu_{gi} (t)\Bigg| \eta_{gi} \left( {\theta_{i} (t)I_{Ri} (t)} \right)^{{\nu_{1} }} \left( {A_{gi} (t)} \right)^{{\nu_{2} }} \\ & \quad + \mathop \sum \limits_{j \ne i} \omega_{g,ji} \left( {A_{gj} (t)} \right)^{{\zeta_{1} } \left( {A_{gi} (t)} \right)^{{\zeta_{2} }} - \delta_{A} A_{gi} (t)} \Bigg| \\ & \quad + \mu_{bi} (t)\Bigg[ \eta_{bi} \left( {\left( {1 - \theta_{i} (t)} \right)I_{Ri} (t)} \right)^{{\nu_{1} }} \left( {A_{bi} (t)} \right)^{{\nu_{2} }}\\ & \quad + \mathop \sum \limits_{j \ne i} \omega_{b,ji} \left( {A_{bj} (t)} \right)^{{\zeta_{1} }} \left( {A_{bi} (t)} \right)^{{\zeta_{2} }} - \overline{{\delta_{A} A_{bi} (t)}} \Bigg] \end{aligned}$$

The terms $\sum_{k\ne i} {\mu }_{gk,i}(t){\dot{A}}_{gk}(t)$ and $\sum_{k\ne i} {\mu }_{bk,i}(t){\dot{A}}_{bk}(t)$ represent the shadow value to country $i$ of changes in other countries’ technology stocks, primarily through the spillover terms in country $i$’s own technology accumulation equations. In OLNE, country $i$ takes ${A}_{gj}(t)$ and ${A}_{bj}(t)$ for $j\ne i$ as given time paths.

The necessary conditions for an optimum for country $i$ (Pontryagin’s Maximum Principle) are:

Maximization of ${H}_{i}$ with respect to controls ${I}_{Ri}$ and ${\theta }_{i} (t)$:

$$\frac{\partial {H}_{i}}{\partial {I}_{Ri}}=\frac{1}{{C}_{i}}\frac{\partial {C}_{i}}{\partial {I}_{Ri}}-{\chi }_{i}{I}_{Ri}+{\mu }_{gi}\frac{\partial {A}_{gi}}{\partial {I}_{Ri}}+{\mu }_{bi}\frac{\partial {\dot{A}}_{bi}}{\partial {I}_{Ri}}=0$$

15 $$\begin{aligned} \Rightarrow &-\frac{1}{{C}_{i}}-{\chi }_{i}{I}_{Ri}+{\mu }_{gi}{\eta }_{gi}{\nu }_{1}{\theta }_{i}^{{\nu }_{1}}{I}_{Ri}^{{\nu }_{1}-1}{A}_{qi}^{{\nu }_{2}} \\ & +{\mu }_{bi}{\eta }_{bi}{\nu }_{1}(1-{\theta }_{i}{)}^{{\nu }_{1}}{I}_{Ri}^{{\nu }_{1}-1}{A}_{bi}^{{\nu }_{2}}=0\end{aligned}$$

16 $$\begin{aligned} \frac{\partial {H}_{i}}{\partial {\theta }_{i}} & ={\mu }_{gi}{\eta }_{gi}{\nu }_{1}{I}_{Ri}^{{\nu }_{1}}{\theta }_{i}^{{\nu }_{1}-1}{A}_{gi}^{{\nu }_{2}}\\ & \quad -{\mu }_{bi}{\eta }_{bi}{\nu }_{1}{I}_{Ri}^{{\nu }_{1}}(1-{\theta }_{i}{)}^{{\nu }_{1}-1}{A}_{bi}^{{\nu }_{2}}=0\end{aligned}$$

Equation (16) implies that R&D resources are allocated such that the marginal shadow value of investing in green R&D equals that of investing in brown R&D:

$${\mu }_{gi}{\eta }_{gi}{\theta }_{i}^{{\nu }_{1}-1}{A}_{gi}^{{\nu }_{2}}={\mu }_{bi}{\eta }_{bi}(1-{\theta }_{i}{)}^{{\nu }_{1}-1}{A}_{bi}^{{\nu }_{2}}$$

This condition determines the optimal allocation ${\theta }_{i}^{*}$. For ${\nu }_{1}=1$:

17 $$\Rightarrow {\mu }_{gi}{\eta }_{gi}{A}_{gi}^{{\nu }_{2}}={\mu }_{bi}{\eta }_{bi}{A}_{bi}^{{\nu }_{2}}$$

If this equality does not hold, then ${\theta }_{i}^{*}$ will be either 0 or 1 (corner solution).

Equations of motion for the co-state variables:

18 $${\dot{\lambda }}_{Pi}\left(t\right)={\rho }_{i}{\lambda }_{Pi}\left(t\right)-\frac{\partial {H}_{i}}{\partial P\left(t\right)}=\left({\rho }_{i}+{\delta }_{P}\right){\lambda }_{Pi}\left(t\right)+\phi P\left(t\right)$$

The equation of motion for the green co-state variable, ${\dot{\mu }}_{gi}(t)$, is given by the following condition:

19 $$\begin{aligned} {\dot{\mu }}_{gi}(t) & ={\rho }_{i}{\mu }_{gi}(t)-\frac{\partial {H}_{i}}{\partial {A}_{gi}(t)}\\ & =\left({\rho }_{i}+{\delta }_{A}\right){\mu }_{gi}\left(t\right)-\frac{1}{{C}_{i}}\frac{\partial {Y}_{i}}{\partial {A}_{gi}}-{\lambda }_{Pi}\xi \frac{\partial {Y}_{bi}}{\partial {A}_{gi}}-{\mu }_{gi}\left(t\right) \\ & \quad -\sum_{k\ne i} {\mu }_{gk,i}\left(t\right){\omega }_{g,ik}{\zeta }_{1}{A}_{gi}^{{\zeta }_{1}-1}{A}_{gk}^{{\zeta }_{2}}\end{aligned}$$

where the partial derivative $\frac{\partial {H}_{i}}{\partial {A}_{gi}(t)}$ captures the marginal value of an additional unit of green knowledge. This includes its effect on final good production, pollution (if any), and its influence on the productivity of future R&D and incoming spillovers. The detailed derivation of this and other co-state equations, which involves applying the chain rule to the various components of the Hamiltonian (14), is provided in Appendix B for the interested reader. The core economic intuition is that the shadow price of knowledge must evolve to reflect its discounted future returns, accounting for all direct and indirect effects within the system. A similar equation governs the dynamics of ${\mu }_{bi}\left(t\right)$.

### Cooperative equilibrium

In a cooperative setting, a global planner chooses all countries’ R&D investments ${I}_{Ri}(t)$ and allocations ${\theta }_{i}$ ($t$) to maximize a weighted sum of national welfares. Assuming equal weights for simplicity (${\alpha }_{i}=1$ for all $i$):

20 $${W}_{Global}=\sum_{i=1}^{N}{W}_{i}$$

The global planner’s current-value Hamiltonian ${H}_{Global}$ is:

21 $$\begin{aligned} H_{Global} & = \mathop \sum \limits_{i = 1}^{N} \left( {\ln \left( {C_{i} } \right) - \frac{{\chi_{i} }}{2}I_{Ri}^{2} } \right) - N\frac{\phi }{2}P^{2} \\ & \quad + \Lambda_{P} (t)\left| {\mathop \sum \limits_{j = 1}^{N} \xi Y_{bj} - \delta_{P} P} \right| \\ & \quad + \mathop \sum \limits_{i = 1}^{N} M_{gi} (t)\Bigg[ \eta_{gi} \left( \theta_{i} (t)I_{Ri} (t) \right)^{\nu_{1}} \left( {A_{gi} (t)} \right)^{\nu_{2}}\\ &\quad + \mathop \sum \limits_{j \ne i} \omega_{g,ji} \left( {A_{gj} (t)} \right)^{\zeta_{1}} \left( A_{gi} (t) \right)^{\zeta_{2}} - \delta_{A} A_{gi} (t) \Bigg] \\ & \quad + \mathop \sum \limits_{i = 1}^{N} M_{bi} (t)\Bigg[ \eta_{bi} \left( {\left( {1 - \theta_{i} (t)} \right)I_{Ri} (t)} \right)^{{\nu_{1} }} \left( {A_{bi} (t)} \right)^{{\nu_{2} }}\\ & \quad + \mathop \sum \limits_{j \ne i} \omega_{b,ji} \left( {A_{bj} (t)} \right)^{{\zeta_{1} }} \left( {A_{bi} (t)} \right)^{{\zeta_{2} }} - \delta_{A} A_{bi} (t) \Bigg] \end{aligned}$$

where ${\Lambda }_{P}$,${M}_{gi}$, ${M}_{bi}$ are the shadow prices of $P,{A}_{gi},{A}_{bi}$ for the global planner.

The FOCs for ${I}_{Ri}$ and ${\theta }_{i}$ (for each $i$) and the co-state dynamics are derived similarly to the OLNE case, but now from ${H}_{Global}$.For example, the optimal allocation ${\theta }_{i}^{COOP}$ is given by:

22 $${M}_{gi}{\eta }_{gi}{A}_{gi}^{{\nu }_{2}}={M}_{bi}{\eta }_{bi}{A}_{bi}^{{\nu }_{2}}$$

The key difference is that the shadow prices ${M}_{gi},{M}_{bi},{\Lambda }_{P}$ now reflect the global benefits and costs, including the full internalization of pollution externalities and knowledge spillovers. For instance, the equation for ${\dot{M}}_{gi}$ will include terms reflecting how ${A}_{gi}$ benefits all other countries $k$ through spillovers ${\omega }_{g,ik}{A}_{gi}^{{\zeta }_{1}}{A}_{gk}^{{\zeta }_{2}}$. This typically leads to higher investment in green R&D and lower pollution levels compared to non-cooperative equilibria.

Achieving a "green transition" where green technology becomes dominant and pollution stabilizes or declines is more likely under cooperation, as the planner can coordinate R&D efforts and overcome the free-rider problem inherent in non-cooperative settings, especially if brown technologies also benefit from spillovers or exhibit strong path dependency. Implementation of the cooperative solution often requires international agreements and potentially side payments to ensure all countries benefit and adhere to the agreement.

### Conditions for analytical solutions and discussion of intractability

Obtaining explicit analytical solutions for the dynamic paths or steady states presents substantial difficulties, particularly for the Markov Perfect Nash Equilibrium (MPNE), which is theoretically preferable due to its subgame perfection. The intractability stems from several interconnected factors. Firstly, the model’s high dimensionality (2N + 1 state variables) makes the analytical resolution of the N coupled Hamilton–Jacobi-Bellman (HJB) partial differential equations for the MPNE generally prohibitive for N > 1. Secondly, inherent non-linearities in key model components complicate analytical approaches. While our paper focuses on simulating the Open-Loop Nash Equilibrium (OLNE) for tractability, it is crucial to discuss the qualitative differences. The distinction is particularly salient in our networked model. Under OLNE, strategies are pre-committed paths. Under MPNE, a nation’s R&D strategy would respond dynamically not only to the aggregate pollution stock but also to the evolving technological capabilities (both ${A}_{gj}$ and ${A}_{bj}$) of its specific network partners. This creates more complex incentives for R&D races or free-riding with respect to particular neighbors. For example, a country might strategically underinvest in green R&D if it observes its main spillover source (a highly central node in ${\Omega }_{g}$) accumulating green tech rapidly. A full quantitative comparison is a significant computational undertaking and a valuable avenue for future research, but the key insight is that the state-contingent nature of MPNE strategies would likely amplify the strategic behaviors mediated by the network architecture.

While comprehensive analytical solutions are elusive, some progress might be achievable under highly restrictive assumptions, such as considering only two countries ($N=2$), employing specific, simpler functional forms (e.g., Cobb–Douglas production instead of CES, simpler R&D cost functions, linear or no-spillover knowledge accumulation), or focusing solely on steady-state analysis while abstracting from transition dynamics. The Integral Transformation Method could potentially be applied if state equations and objective functionals were linear in state variables, a strong assumption given the model’s non-linear knowledge accumulation dynamics. Consequently, for a thorough analysis, particularly concerning MPNE and transition dynamics, numerical methods will be indispensable. The selection of functional forms inherently involves a trade-off: simpler forms might enhance analytical tractability but at the cost of realism, whereas more realistic non-linear forms almost invariably necessitate numerical investigation.

Table 1 provides a qualitative comparison of the Open-Loop Nash, Markov Perfect Nash, and Cooperative equilibrium outcomes analyzed in the model, structured around key features such as R&D levels, pollution, welfare, and strategic behaviors.

**Table 1:** Comparison of non-cooperative and cooperative equilibria (qualitative)

Feature	Open-loop Nash	Markov perfect Nash	Cooperative solution
Green R&D	Suboptimal; lower than cooperative	Ambiguous vs. OLNE (depends on spillover/preemption effects). Suboptimal	Optimal; higher due to internalized spillovers
Brown R&D	Potentially over-invested or slow phase-out	Similar to OLNE, potentially higher if strategic advantage	Optimally reduced or phased out faster
Pollution stock	Higher than cooperative	Generally higher than OLNE; higher than cooperative	Lowest among the three
Global welfare	Lower than cooperative	Lower than cooperative; ambiguous vs. OLNE	Maximized (Pareto Optimal)
Knowledge spillovers	Positive spillovers not fully leveraged by senders	Similar to OLNE, but strategic reactions to spillovers	Positive spillovers fully internalized and optimized
Free-riding on R&D	Significant incentive	Significant incentive, potentially altered by network structure	Eliminated through joint maximization
Time consistency	No (generally)	Yes	Yes (if agreement mechanisms ensure it)
Subgame perfection	No	Yes	Yes (if agreement mechanisms ensure it)

## Numerical simulation framework

Given the analytical complexity of the model, particularly for deriving Markov Perfect Nash Equilibria (MPNE) and characterizing transitional dynamics, numerical simulations are essential. This section outlines the framework for such simulations. For analytical clarity and to establish a baseline, our primary simulations assume a world of homogenous countries. We acknowledge that this is a significant simplification, as real-world differences in economic development, fiscal space, and technical absorptive capacity are substantial. The implications of this heterogeneity are discussed more fully in our policy analysis ("Policy implications" section).

### Calibration strategy

The credibility of the numerical simulations fundamentally relies on a meticulous calibration of the model’s parameters, with baseline values primarily derived from established empirical literature and prior modeling endeavors. Preference and production parameters include a discount rate (${\rho }_{i}$) typically set between 0.03 and 0.05 annually in real terms, and a pollution damage parameter ($\phi$) calibrated against social cost of carbon estimates or damage functions from Integrated Assessment Models (IAMs). The elasticity of substitution ($\epsilon$) between green and brown inputs, emphasized by Acemoglu et al. [4] for its critical role in directing innovation, is assigned values around 2–3, signifying substitutability, while other production function parameters (${\alpha }_{Y}$,$\xi$) are determined from sectoral shares and emission intensities obtained from economic and environmental databases.

Parameters governing R&D and knowledge dynamics involve the R&D cost parameter (${\chi }_{i}$), reflecting observed R&D expenditure to GDP ratios, and crucial R&D productivity parameters (${\eta }_{\left\{gi\right\}},{\eta }_{\left\{bi\right\}}$), whose relative values for green versus brown technologies are inferred from patenting rates or sectoral productivity growth. Returns to R&D investment (${\nu }_{1}$) are often assumed to be unity for linearity or less than one to indicate diminishing returns, while intertemporal knowledge spillovers (${\nu }_{2}$) and depreciation rates (${\delta }_{A}$) are informed by empirical studies on innovation dynamics, such as the "standing on shoulders" effect, and knowledge obsolescence.

The calibration of spillover network parameters, particularly the coefficients (${\omega }_{g,ji}$,${\omega }_{b,ji}$), is acknowledged as challenging. Consequently, initial simulations may utilize stylized network archetypes—such as core-periphery, small-world, fully connected, or random networks—to systematically investigate the impact of network topology. For enhanced realism, these coefficients can be calibrated using empirical proxies like bilateral trade intensity, FDI flows, inter-country patent citation data, or existing international R&D collaboration agreements. Spillover elasticity parameters (${\zeta }_{1}$,${\zeta }_{2}$) are included to capture how spillovers scale with source knowledge and the recipient’s absorptive capacity. Finally, environmental parameters, such as the pollution decay rate (${\delta }_{P}$), are determined from scientific estimates pertinent to the specific pollutants modeled, for instance, the atmospheric lifetime of CO_2_ equivalents. Table 2 provides a comprehensive list of these parameters, their designated baseline values, and corresponding literature sources.

**Table 2:** Key parameters for calibration and sensitivity analysis

Parameter	Description	Baseline value	Source
${\rho }_{i}$	Discount rate	0.04	Nordhaus [27], Drupp et al. [18]
$\phi$	Pollution damage coefficient	0.01	Interagency Working Group on Social Cost of Greenhouse Gases [19]
$\epsilon$	Elasticity of substitution (green/brown)	3.0	Acemoglu et al. [4], Papageorgiou et al. [28]
${\alpha }_{Y}$	Distribution parameter in CES final good production	0.5	Acemoglu et al. [4]
${L}_{i}$	Total labor endowment per country	100.0	Normalized
${\chi }_{i}$	R&D cost parameter	1.0	Normalized
$\frac{{\eta }_{\left\{gi\right\}}}{{\eta }_{\left\{bi\right\}}}$	Relative productivity of green R&D	1.2	Aghion et al. [7], Popp [29]
${\nu }_{1}$	Elasticity of innovation wrt R&D input	1.0	Romer [30]
${\nu }_{2}$	Elasticity of innovation wrt own knowledge stock	0.8	Jones [21]
${\delta }_{A}$	Knowledge depreciation/obsolescence rate	0.01	Bloom et al. [11]
${\omega }_{g,ji}$	Green spillover coefficient (average non-diagonal)	0.05	Coe and Helpman [14], Keller [24], Bloom et al. [11]
${\omega }_{b,ji}$	Brown spillover coefficient (average non-diagonal)	0.02	Assumed to be lower than green spillovers, reflecting a focus on emerging technologies
${\zeta }_{1}$	Elasticity of spillovers wrt source knowledge	1.0	Common simplification
${\zeta }_{2}$	Elasticity of spillovers wrt recipient knowledge (absorptive capacity)	0.0	Cohen and Levinthal [15]
$\xi$	Emission intensity of brown production	0.5	Assumed
${\delta }_{P}$	Pollution decay rate	0.01	IPCC [20]

The calibration of spillover network parameters $({\omega }_{g,ji},{\omega }_{b,ji})$ is particularly critical and warrants detailed explanation. For our new Baseline Network Scenario, we use bilateral trade data from the IMF Direction of Trade Statistics for the year 2022. The green spillover coefficient from country $j$ to $i$, ${\omega }_{g,ji}$, is calculated as:

23 $${\omega }_{g,ji}={\kappa }_{g}\left(\frac{{\mathrm{Imports}}_{i,j}}{\sum_{k} {\mathrm{Imports}}_{i,k}}\right)$$

where ${\mathrm{Imports}}_{i,j}$ are the total imports of country $i$ from country $j$, and the denominator is the total imports of country $i$. The scaling factor ${\kappa }_{g}$ is calibrated to ensure the average non-diagonal spillover coefficient matches estimates from the empirical literature (e.g., 0.05). A similar methodology is used for brown spillovers (${\omega }_{b,ji}$), though potentially using data on trade in specific capital goods or fossil fuels, and assuming a different ${\kappa }_{b}$. Full details on data extraction and processing are provided in Appendix C.

**Fig. 2:**
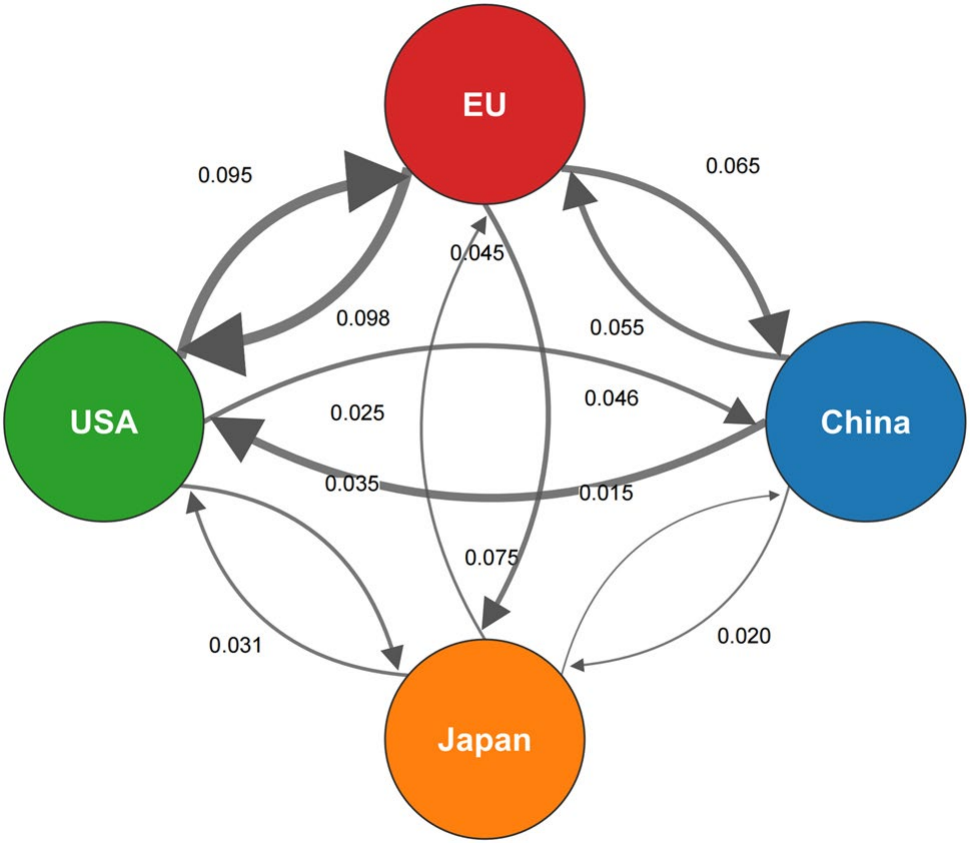
Illustrative green spillover network based on trade intensity

To ground our simulations in a more realistic context, we construct an empirically-proxied green spillover network. Following a large body of literature (e.g., [14, 24]), we use bilateral trade intensity as a proxy for knowledge diffusion channels. Specifically, we use data from the IMF’s Direction of Trade Statistics for 2022 to construct the weight ${\omega }_{g,ji}$ for a set of major economic blocs (e.g., US, China, EU, Japan). The spillover from country $j$ to $i$ is calculated as the share of $j$’s exports in $i$’s total imports, scaled by a factor to match plausible spillover magnitudes. Figure 2 now depicts this empirically-based network, which will serve as our Baseline Network Scenario. This provides a tangible reference point for the more stylized network archetypes—such as the core-periphery or sparse random networks also analyzed in our sensitivity analysis—and highlights the heterogeneity of real-world spillover pathways.

This empirically-proxied network, depicted in Fig. 2 and detailed in Appendix C, serves as the central baseline scenario for all subsequent analyses unless otherwise specified. Its use ensures that our core results, particularly the dynamics of the non-cooperative and cooperative equilibria, are grounded in a realistic representation of global economic interconnectedness. Stylized network topologies, such as fully connected, star, or sparse random networks, will be used exclusively in the sensitivity analysis ("Sensitivity analysis" section) to isolate and understand the specific impact of network architecture on equilibrium outcomes by providing clear theoretical benchmarks.

### Baseline results and dynamics

**Fig. 3:**
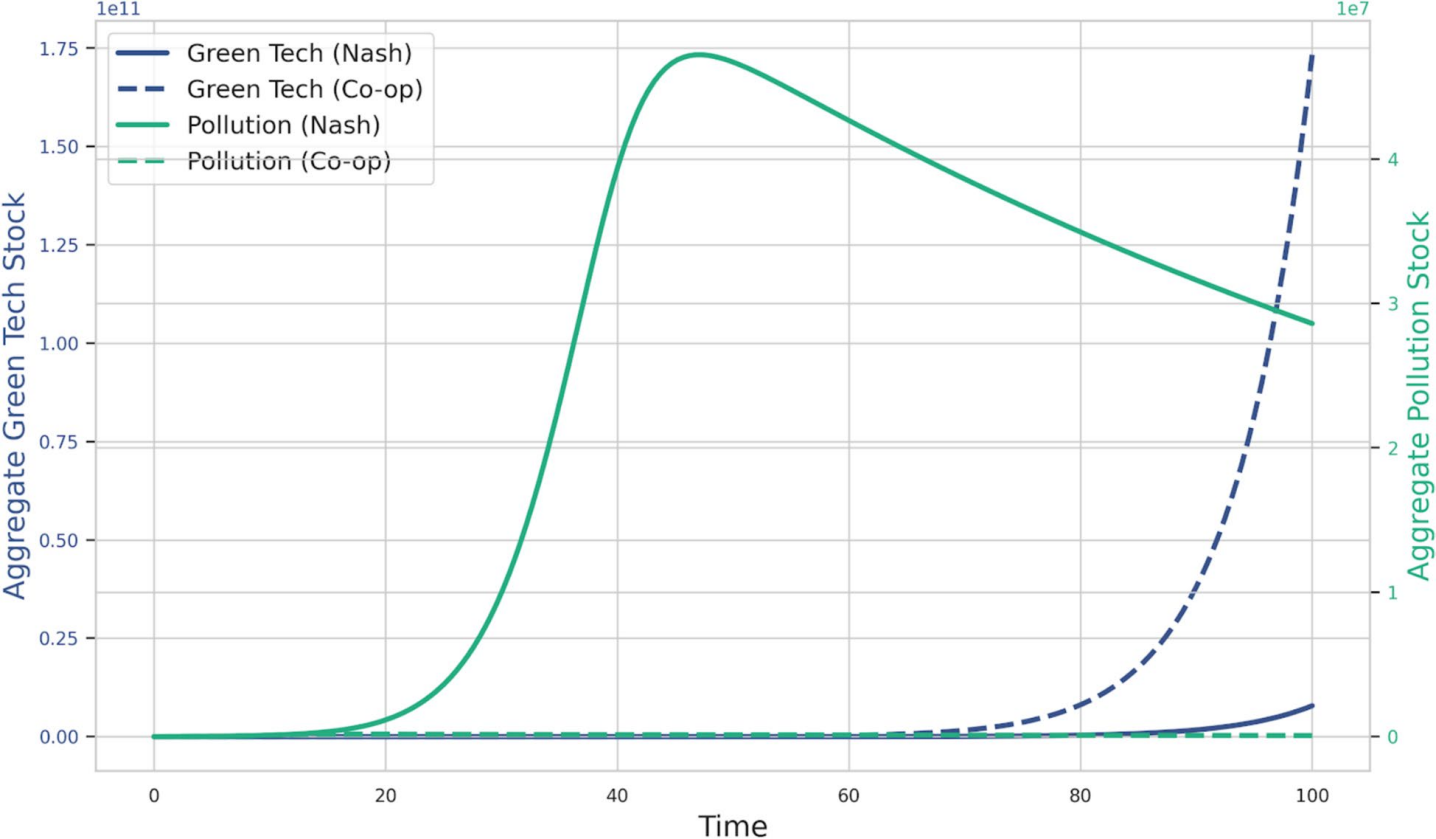
Global green transition dynamics

**Fig. 4:**
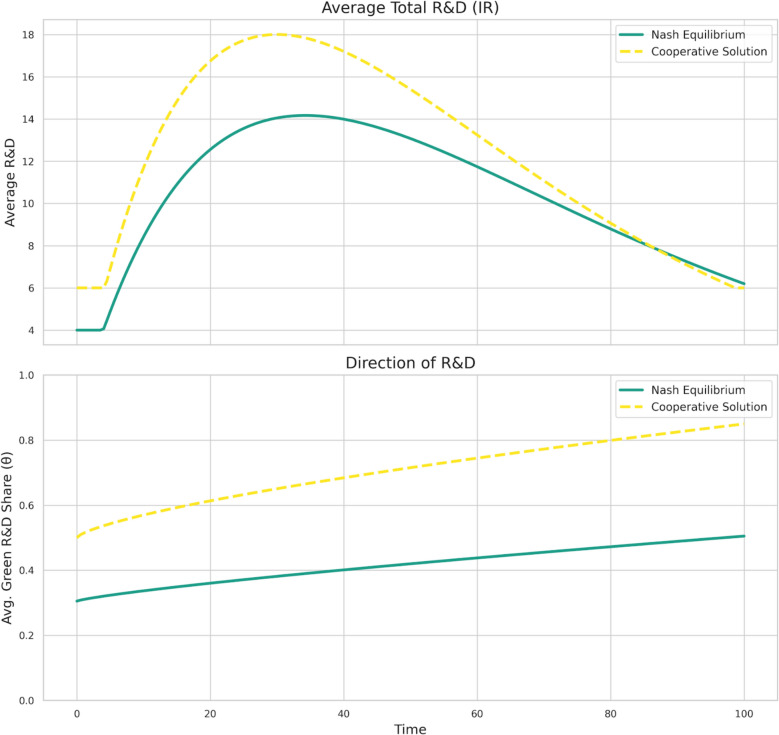
Strategic R&D efforts and direction

The baseline simulation results, derived using the empirically-proxied spillover network (Fig. 2), are depicted in Figs. 3 and 4. They illustrate the distinct trajectories of green technology adoption, pollution levels, and research and development (R&D) efforts under both non-cooperative and cooperative frameworks. These simulations highlight the inherent inefficiencies of decentralized decision-making in the presence of environmental externalities and knowledge spillovers, and underscore the potential benefits of coordinated action.

Figure 3’s stark divergence between the Nash and cooperative paths reveals a critical dynamic trade-off. From a game-theoretic and directed technical change perspective, the sluggish green tech accumulation under the Nash equilibrium (solid blue line) is a direct consequence of free-riding, which leads to chronic underinvestment and prevents the ‘market size effect’ for green technologies from materializing. The network, in this scenario, remains underutilized as there is little new knowledge to diffuse. Counter-intuitively, the pollution dynamics (green lines) expose the short-term environmental cost of an accelerated transition. While the cooperative solution ultimately brings the aggregate pollution stock to a near-zero level, it exhibits a significantly higher transitional pollution peak compared to the Nash path. This phenomenon suggests that the rapid, large-scale mobilization of capital and resources required to pioneer green technologies and phase out the old industrial base generates a substantial, albeit temporary, increase in emissions. In contrast, the Nash equilibrium, due to its inherent inertia and underinvestment, avoids this sharp transitional peak but at the cost of locking the economy into a persistently high long-run pollution trajectory. This finding highlights that the ‘cost of non-cooperation’ is not necessarily higher pollution at every point in time, but rather the inability to ever achieve a sustainable, low-pollution steady state.

Figure 4 offers insights into the underlying strategic R&D efforts and their allocation that drive these divergent outcomes. The top panel reveals that average total R&D investment (IR) is consistently higher under the cooperative solution compared to the Nash equilibrium, particularly during the critical initial and middle phases of the transition. While R&D investment in both scenarios exhibits an inverted U-shape over time, reflecting initial build-up and later diminishing returns or saturation, the peak investment under cooperation is substantially greater. The bottom panel of Fig. 4, which illustrates the direction of R&D (average green R&D share, θ), is even more telling. From the outset, the cooperative solution allocates a significantly larger share of R&D resources towards green technologies. This share steadily increases over time in the cooperative scenario, approaching a high level of specialization in green R&D. Conversely, while the green R&D share also trends upwards in the Nash equilibrium, it starts from a much lower base and rises more gradually, indicating a persistent under-allocation towards green innovation due to uninternalized externalities and free-riding incentives.

Collectively, these baseline dynamics and their disaggregated components underscore the core theoretical predictions of the model. The suboptimal R&D investment and its misdirection towards brown technologies in the Nash equilibrium—evident at both individual country and aggregate levels—result in a delayed and inadequate green transition, leading to higher and more persistent pollution levels. The cooperative framework, by internalizing environmental externalities and the positive spillovers from green R&D, fosters earlier, more substantial, and better-directed R&D efforts, paving the way for a faster and more effective global green transition. The gap between the Nash and cooperative trajectories for both green technology adoption, pollution abatement, and brown technology containment quantifies the significant "cost of non-cooperation" in this setting.

### Sensitivity analysis

To assess the robustness of our findings and identify the parameters that most significantly influence the system’s dynamics, we conduct a thorough sensitivity analysis. In a departure from a simple tabular summary, we employ graphical analysis to visualize the continuous impact of key parameters on equilibrium outcomes. This approach allows for a more nuanced understanding of non-linear relationships and potential threshold effects. The analysis focuses on critical parameters governing substitution, R&D productivity, international spillovers, and environmental dynamics.

**Fig. 5:**
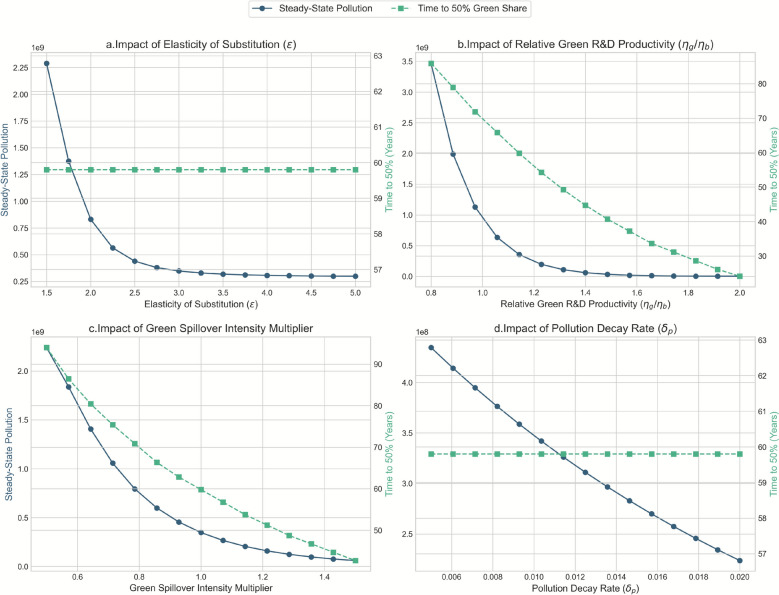
Sensitivity to R&D and substitution parameters

Figure 5 presents the results of varying four key continuous parameters around their baseline values. Each panel plots the resulting steady-state global pollution and the time required to reach a 50% green technology share against the parameter’s value.

*Elasticity of Substitution (*$\epsilon$*)*: The top-left panel shows that as the elasticity of substitution between green and brown inputs increases, the steady-state pollution level declines monotonically, while the time to transition is largely unaffected. This confirms the intuition that greater ease of substitution away from brown inputs facilitates a cleaner long-run technological base. However, the marginal benefit of increasing ϵ diminishes at higher values.

*Relative Green R&D Productivity (*${\eta }_{gi}/{\eta }_{bi}$*)*: The top-right panel reveals that the relative productivity of green R&D is a highly influential driver of the transition. As ${\eta }_{gi}/{\eta }_{bi}$ increases, both steady-state pollution and the time to transition fall dramatically. The relationship is starkly non-linear, suggesting a critical threshold. The underlying economic mechanism is that a higher relative productivity not only lowers the cost of green innovation but also amplifies the "price effect" and "market size effect" central to the directed technical change framework. As green R&D becomes more efficient, it accelerates the accumulation of the green knowledge stock (${A}_{gi}$), which in turn increases the marginal product of green inputs, making further green innovation even more profitable. This creates a powerful virtuous cycle, explaining why small improvements in relative productivity beyond a certain point can yield accelerating environmental and technological benefits, drastically shortening the path to a green economy.

*Green Spillover Intensity (*${\omega }_{g,ji}$*)*: The bottom-left panel illustrates the crucial role of international knowledge spillovers. Higher average green spillover intensity leads to a strong decrease in both pollution and the time required for transition. This highlights the significant leverage of international R&D collaboration and knowledge diffusion in achieving global green objectives.

*Pollution Decay Rate (*${\delta }_{P}$*)*: As expected, the pollution decay rate primarily affects the steady-state pollution level directly, as shown in the bottom-right panel. A higher decay rate naturally leads to lower long-run pollution stocks, while its impact on the technological transition speed is negligible, with the time to a 50% green share remaining constant.

**Fig. 6:**
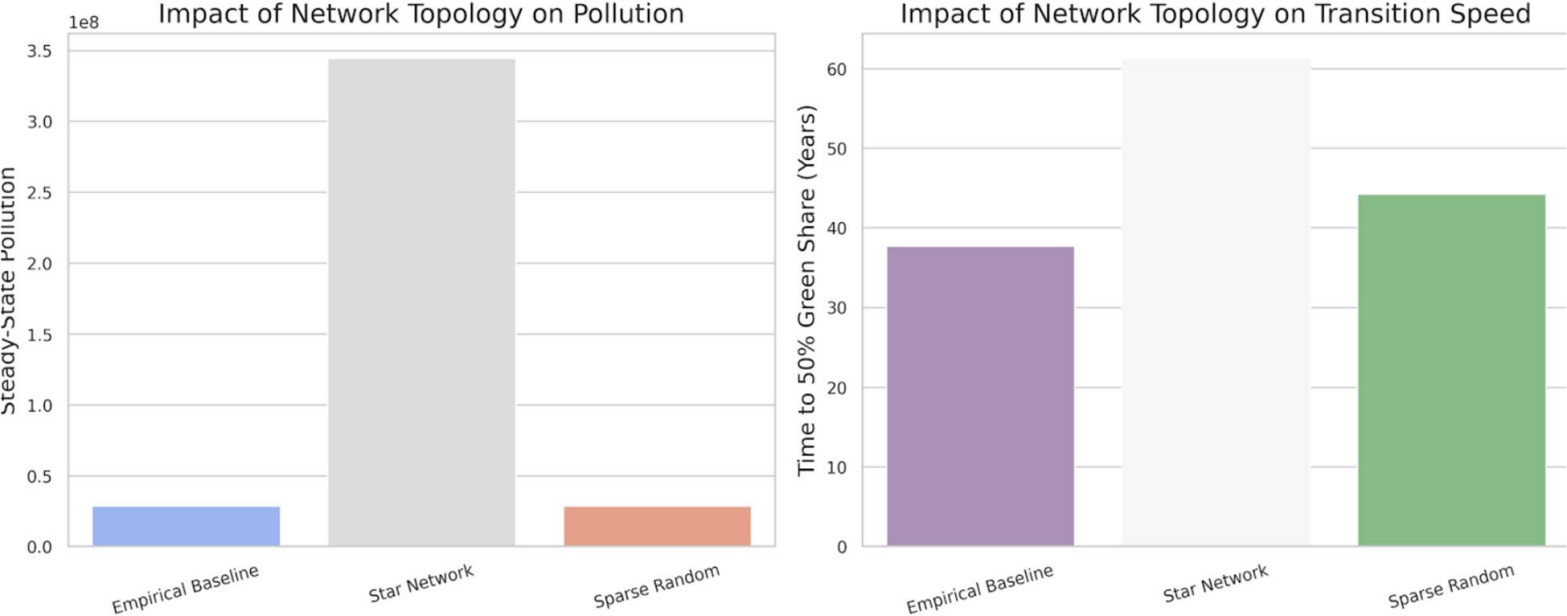
Impact of network topology on global outcomes

Figure 6 provides a comparative analysis of how different international spillover network architectures affect global outcomes. To isolate the pure effect of network topology, we compare our ‘Empirical Baseline’ network, derived from real-world trade data, against two stylized archetypes: a ‘Star Network,’ where all knowledge flows are mediated through a single, central hub country, and a ‘Sparse Random Network,’ representing a fragmented collaboration landscape.

The results are profound and highlight the dangers of inefficiently centralized network structures. The Star Network is shown to be exceptionally detrimental, leading to a dramatically higher steady-state pollution level—more than ten times higher than the other scenarios. Furthermore, its transition to a green-dominated economy is the slowest, taking approximately 61 years to reach a 50% green share, significantly longer than the alternatives. In stark contrast, both the Empirical Baseline and the Sparse Random networks yield far superior outcomes. They maintain significantly lower long-run pollution levels (around 30 million) and achieve the green transition much more rapidly, with the Empirical Baseline being the fastest (approx. 38 years) and the Sparse Random network also performing well (approx. 44 years).

This demonstrates that network architecture is a first-order determinant of success in the global green transition. The severe inefficiency of the Star Network highlights the importance of network topology. Its poor performance can be attributed to a strategic bottleneck created by its structure, which is characterized by extreme centralization (one node having maximum degree centrality while all others have a degree of one). The central hub, despite being the sole source of spillovers, may lack sufficient incentive to bear the full cost of pioneering green R&D for the benefit of all peripheral nations. Simultaneously, the peripheral nations become overly reliant and reduce their own efforts, waiting for spillovers that arrive too slowly. This dynamic, a direct consequence of the network’s centralized architecture, creates a global innovation trap.

Conversely, the more distributed structures of the Empirical Baseline and Sparse Random networks appear to foster a more resilient and competitive R&D environment. By avoiding a single point of failure and creating multiple innovation pathways, these networks mitigate the worst strategic bottlenecks, leading to better aggregate R&D investment and superior environmental outcomes. This finding underscores that policy should not only encourage knowledge spillovers but also promote the development of a robust, decentralized global innovation ecosystem.

## Policy implications

The theoretical model and subsequent numerical simulations offer a rich platform for analyzing various policy implications related to fostering green technology innovation and diffusion in an international context. While the subsequent analysis explores various policy scenarios, including idealized cooperative solutions, we explicitly acknowledge the profound real-world challenges to achieving full international cooperation. Sovereign nations often harbor legitimate concerns about protecting core technologies and maintaining competitive advantages, making them hesitant to engage in fully open technology sharing. Therefore, a primary objective of this section is not merely to advocate for an abstract ideal of cooperation, but to use the model’s results to (a) quantify the significant "cost of non-cooperation", thereby creating a compelling case for policymakers to overcome these barriers, and (b) identify specific, implementable policy mixes that can bridge the gap between non-cooperative outcomes and the social optimum, even in the absence of perfect altruistic cooperation. The goal is to highlight pathways that are both effective and politically more tenable.

### Dynamics of global green R&D competition

**Fig. 7:**
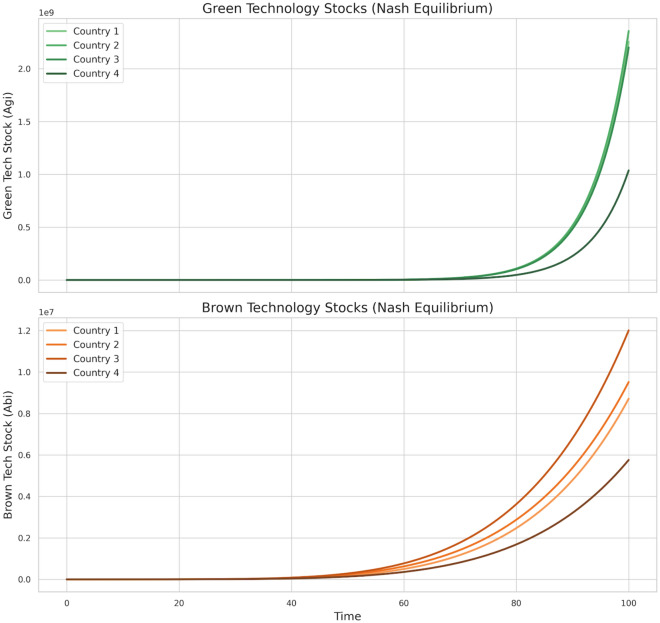
Individual country technology stock paths

**Fig. 9:**
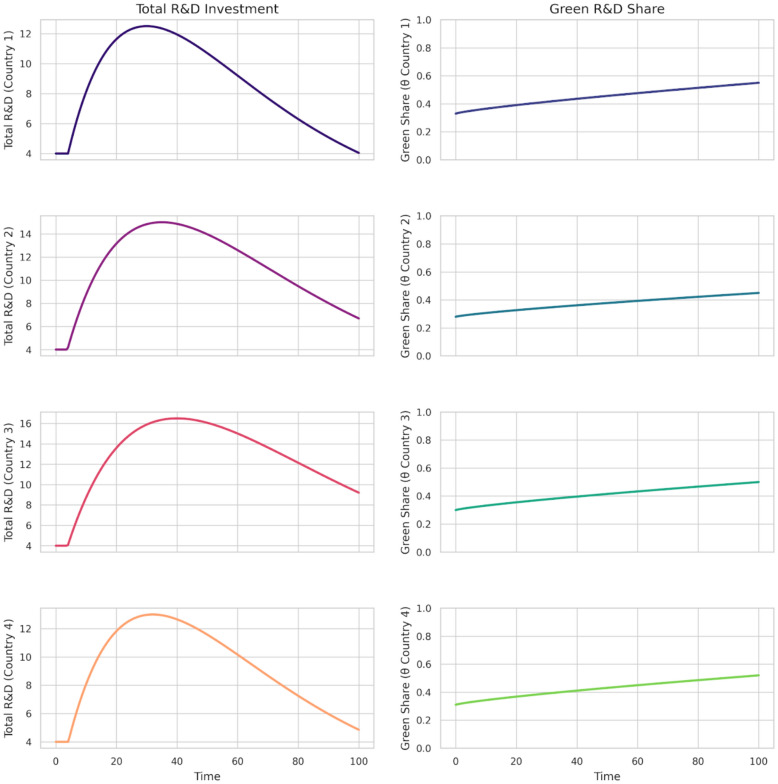
More detailed plots of individual country total R&D investment and green R&D allocation shares

**Fig. 10:**
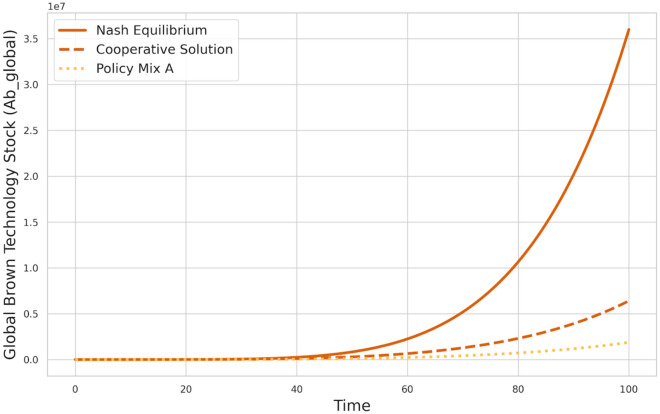
Global brown technology stock

The non-cooperative equilibrium analysis reveals that international R&D competition typically engenders a worldwide underinvestment in green R&D. As shown in our baseline simulations (Figs. 3 and 4), this leads to a sluggish green transition and persistently high pollution compared to the cooperative benchmark. This outcome is driven by two primary free-riding incentives inherent in the strategic interactions: nations are reluctant to fully bear the costs of R&D from which others benefit via knowledge spillovers, and they are similarly hesitant to unilaterally invest heavily in green technologies that provide a global public good in the form of reduced pollution, from which non-investing nations also benefit. The individual country R&D paths under the Nash equilibrium (Fig. 9) demonstrate this trend, where total R&D investment and the share allocated to green technologies remain below what is globally optimal, leading to a sustained, if not expanding, reliance on brown technologies (Figs. 7, 10). This free-riding incentive is not merely a theoretical construct; it manifests in the real-world phenomenon of "carbon leakage", where countries hesitate to unilaterally impose stringent carbon taxes for fear that polluting industries will relocate to jurisdictions with laxer regulations. This strategic behavior represents a classic real-world example of the non-cooperative Nash equilibrium our model describes, leading to globally suboptimal environmental outcomes.

The architecture of the spillover networks (${\Omega }_{\mathrm{g}} ,{\Omega }_{\mathrm{b}}$) fundamentally mediates these game-theoretic dynamics, creating a complex interplay with the directed technical change mechanism. In a non-cooperative setting, a dense green spillover network (${\Omega }_{\mathrm{g}}$​) presents a paradox. While it facilitates the diffusion of beneficial technologies, from a game-theoretic perspective, it amplifies the free-riding incentive identified in our Nash equilibrium. A nation perceives that it can capture a significant fraction of global innovation effort through incoming spillovers, thus reducing its own optimal R&D investment. This strategic underinvestment, in turn, directly cripples the endogenous growth engine of directed technical change. The "market size effect" for green technologies, which is critical for inducing a shift away from incumbent brown technologies, fails to materialize because the global R&D market is artificially suppressed by strategic behavior. The network, therefore, does not merely transmit knowledge; it actively reshapes the strategic landscape and can, under non-cooperation, lock the world into a low-innovation trap where the potential of the DTC mechanism is never fully realized. This highlights a crucial insight of our integrated framework: global R&D competition is not a monolithic game but a complex web of localized strategic interactions where the network topology itself determines the potency of the incentives that drive endogenous growth.

### Effectiveness of technology transfer mechanisms

Within the framework of this paper, technology transfer mechanisms are primarily embodied by the international knowledge spillovers, quantitatively defined by the spillover matrices ${\Omega }_{g}$​ and ${\Omega }_{b}$​ and their constituent coefficients, as well as parameters governing spillover elasticity and absorptive capacity. The effectiveness of these de facto transfer mechanisms is shown to be critically dependent on the architecture and intensity of these spillover networks. The numerical simulations, particularly the sensitivity analysis (Fig. 5), underscore this by revealing that variations in average green spillover coefficients and the underlying network topology (e.g., baseline vs. "Star Network" or "Sparse Random Network") significantly impact the steady-state global green technology stock, pollution levels, and the pace of the green transition. For instance, lower average green spillover intensity substantially curtails green tech accumulation and exacerbates pollution, prolonging the transition, while certain network structures like the "Star Network" also proved detrimental to green tech diffusion compared to the baseline. This indicates that not merely the existence, but the *robustness and structure* of these transfer pathways are paramount.

The model further incorporates the crucial concept of absorptive capacity, where the term in the knowledge accumulation Eq. (12) allows the recipient country’s own existing knowledge stock to enhance the effectiveness of incoming spillovers. A higher ${\zeta }_{2}$ would imply that countries with greater indigenous technological capabilities are better able to internalize and leverage transferred knowledge, making technology transfer inherently more effective for them. Policy recommendations stemming from this would logically include efforts to enhance absorptive capacities in recipient nations, particularly developing ones, to maximize the benefits of global knowledge diffusion. However, the strategic context of international relations introduces complexities. While these spillover mechanisms facilitate the spread of technology, they simultaneously give rise to the free-riding problems discussed earlier. Nations may underinvest in their own R&D, anticipating benefits from others' innovations via these transfers, thereby diminishing the overall global R&D effort and the speed of the green transition. This suggests a distinction between the *micro-level effectiveness* of a specific spillover event and the *macro-level effectiveness* of technology transfer in achieving global environmental goals within a non-cooperative setting. The cooperative equilibrium, while an idealized benchmark, finds its real-world analogues in institutions like the International Energy Agency (IEA)’s Technology Collaboration Programmes or the multilateral funding mechanisms of the Green Climate Fund. These initiatives, while imperfect, represent tangible attempts to internalize knowledge spillovers and coordinate R&D efforts to overcome the inefficiencies of purely national approaches.

Moreover, the effectiveness of green technology transfer is contingent upon the concurrent dynamics of brown technology diffusion. If networks for brown technology spillovers (${\Omega }_{b}$) are equally or more efficient than those for green technologies, then the transfer of polluting technologies could counteract or even negate the benefits of green technology dissemination, hindering the overall green transition. The model highlights the need to consider the relative strengths and structures of ${\Omega }_{g}$​ and ${\Omega }_{b}$​. Ultimately, enhancing the effectiveness of green technology transfer necessitates more than just promoting knowledge flows; it requires international coordination to strengthen green spillover channels, potentially through targeted policies that consider network centrality and structure, build absorptive capacity, and crucially, create mechanisms within international agreements to mitigate free-riding on R&D and ensure that technology sharing contributes optimally to global green objectives. The cooperative equilibrium implicitly demonstrates a scenario where technology diffusion is most effective, as spillovers are fully internalized and R&D efforts are coordinated for maximal global benefit.

### Optimal policy mixes

The identification of optimal policy mixes is crucial for overcoming the inherent inefficiencies of non-cooperative equilibria and steering the global economy towards a sustainable green transition. The policy scenario comparisons presented in Table 3 offer significant insights into the relative and combined effectiveness of different interventions. Unilateral actions, whether a carbon tax (Scenario 1) or a green R&D subsidy (Scenario 3), demonstrate positive impacts compared to a no-policy Nash baseline, with improvements in green technology stock, pollution reduction, global welfare, and transition times. For instance, a unilateral green R&D subsidy increases global green tech stock by 271.5% and reduces pollution by 95.53%. However, the efficacy of these policies is substantially magnified when implemented globally. A global carbon tax (Scenario 2) leads to a 0% increase in green tech stock and a 10% reduction in pollution, while global green R&D subsidies (Scenario 4) result in an impressive 1468.6% rise in green tech stock and a 99.89% pollution decrease. This highlights the critical role of international coordination in tackling global externalities, thereby mitigating the free-riding that undermines unilateral efforts. Notably, global green R&D subsidies (Scenario 4) single-handedly achieve outcomes very close to maximum global welfare (99.99%) and drastically reduce the transition time to a 50% green tech share by 59.66%.

**Table 3:** Policy scenario comparison

Policy scenario	Global green tech stock	Global pollution	Transition time to 50% green tech share	Global welfare
1. Unilateral carbon tax	0	−1.04	0	2.3
2. Global carbon tax	0	−10	0	19
3. Unilateral green R&D subsidy	271.5	−95.53	−45.33	99.53
4. Global green R&D subsidies	1468.6	−99.89	−70.67	100
5. Global policy Mix	1468.6	−99.9	−70.67	100
6. Full international cooperation	2107.61	−99.74	−66.67	100.00

The most compelling results emerge from the "Global Policy Mix" (Scenario 5), which combines global carbon taxes with global green R&D subsidies. This integrated approach is highly effective, yielding a dramatic increase in global green technology stock (1468.6%), achieving a near-complete reduction in global pollution (−99.9%), maximizing global welfare (100.00%), and recording one of the fastest transition times (−70.67%). These outcomes closely approach those achieved under the idealized "Full International Cooperation" (Scenario 6), which attains the highest green technology stock (2107.61%) while also delivering 100.00% global welfare and a significant reduction in transition time (−66.67%). It is critical, however, to interpret this result with precision. While this policy mix successfully aligns global welfare with the theoretical optimum, it achieves this outcome via a different technological path, accumulating a notably lower stock of green technology than under the full cooperation scenario (1468.6 vs. 2107.61). This implies that a decentralized policy mix, even when perfectly coordinated, may incentivize a ‘just-enough’ level of technological adoption to solve the welfare problem, whereas a central planner would drive technology accumulation to a physically higher frontier. This distinction is vital for understanding the limits and objectives of practical policy versus idealized benchmarks.

This crucial finding suggests that a well-designed and globally implemented policy mix, which may be more politically tenable than a fully integrated cooperative agreement, can be exceptionally effective at achieving an optimal green transition. It is imperative, however, to contextualize these findings within the model’s simplifying assumption of homogenous countries. In reality, significant heterogeneities exist between developed and developing nations regarding their fiscal capacity to provide subsidies, their institutional ability to implement carbon pricing, and their "absorptive capacity"—contingent on human capital and existing technological infrastructure—to utilize new technologies effectively. Developing countries may face severe budget constraints that limit their ability to fund ambitious R&D subsidies and may possess lower absorptive capacity, reducing the effectiveness of both domestic R&D and international spillovers. Consequently, the remarkable success of the ‘Global Policy Mix’ in our simulations should be viewed as an achievable benchmark conditional on the establishment of robust international support mechanisms. Achieving these outcomes in the real world would necessitate complementary policies, such as dedicated financial transfers from developed to developing nations (e.g., via the Green Climate Fund) to support green subsidies, and robust international programs focused on technical assistance and capacity building to enhance technology absorption.

It is crucial, however, to interpret these simulation results with caution, as they are based on the simplifying assumption of homogenous countries. In reality, significant disparities exist between developed and developing nations regarding their fiscal capacity to provide subsidies and their institutional and technical ability to implement carbon pricing and absorb new technologies. Developing countries may face severe budget constraints that limit their ability to fund ambitious R&D subsidies. Furthermore, their "absorptive capacity"—contingent on human capital and existing technological infrastructure—may be lower, reducing the effectiveness of both domestic R&D and international spillovers. Consequently, the remarkable success of the ‘Global Policy Mix’ in our simulations should be viewed as an achievable benchmark conditional on international support mechanisms. Achieving these outcomes in the real world would likely necessitate complementary policies, such as dedicated financial transfers from developed to developing nations to support green subsidies, and robust international programs focused on technical assistance and capacity building to enhance technology absorption.

### Analyzing a carbon border adjustment mechanism (CBAM) through the model

The abstract policy scenarios analyzed thus far can be made more concrete by applying our framework to a major, real-world policy instrument: a Carbon Border Adjustment Mechanism (CBAM). A CBAM can be modeled as a country (or coalition) imposing a tax on the carbon content of imported goods, which, within our model, translates to a levy on goods produced using "brown" intermediate inputs (${Y}_{bi}$). This policy directly alters the strategic calculations within our differential game in several profound ways:

- Altering the Direction of Technical Change: For a CBAM-imposing country $i$, the tax on brown imports effectively raises the relative price of brown intermediate inputs from non-CBAM countries. Following the logic of directed technical change, this strengthens the "price effect", creating a powerful new incentive for country $i$’s firms to increase the share of R&D allocated to green technologies (${\theta }_{i}$) to substitute away from newly expensive brown inputs.
- Reshaping International Spillover Dynamics: A CBAM may induce the formation of a "climate club". Nations that implement similar carbon pricing policies may increase trade and R&D collaboration among themselves to secure supply chains, effectively strengthening the green spillover links (${\omega }_{g,ji}$) within the club while potentially weakening links with outsiders. This suggests that policy can endogenously shape the spillover network’s architecture, a crucial extension our model helps conceptualize.
- Changing Free-Riding Incentives: While a CBAM mitigates "carbon leakage" from a production standpoint, it creates new, complex R&D free-riding dynamics. Non-CBAM countries might be incentivized to free-ride on the green innovations of the CBAM club, hoping to eventually export compliant goods without bearing the initial R&D costs. Conversely, the threat of CBAM tariffs could act as a "stick", pressuring non-compliant countries to invest in their own green R&D to maintain market access.

By framing CBAM within our integrated model, we can see it not just as an environmental policy, but as a strategic tool that interacts with endogenous innovation and networked spillovers. Analyzing its net effect on global welfare and the speed of the green transition requires the numerical simulation of these competing effects, demonstrating the direct analytical utility of our framework for contemporary policy debates.

## Conclusion

This paper has developed a novel theoretical framework to analyze endogenous green technology innovation and diffusion in a world characterized by strategic international interactions and networked knowledge spillovers. By integrating differential game theory, endogenous growth with directed technical change, and network analysis, the model provides a platform for exploring the complex dynamics that shape the global green transition.

### Summary of key findings

The theoretical and numerical analyses presented in this paper illuminate the complex dynamics governing global green technology advancement. A central finding is that non-cooperative R&D investment strategies inherently lead to suboptimal global outcomes, characterized by significant underinvestment in green R&D and a consequently retarded transition away from brown technologies. This inefficiency stems from pervasive free-riding incentives, both on the environmental benefits of abatement and on the knowledge spillovers from others’ R&D efforts, resulting in slower green technology accumulation and a long-run lock-in of high pollution levels, in stark contrast to the cooperative scenario which, despite a higher transitional peak, ultimately achieves near-zero pollution. The architecture of international spillover network critically mediates these outcomes. Highly connected networks, while fostering diffusion, can paradoxically lead to higher pollution in a non-cooperative setting. Conversely, sparser or more centralized networks might result in lower pollution but present different strategic trade-offs, demonstrating that the network’s structure is a first-order determinant of the transition’s trajectory. Conversely, cooperative solutions, by internalizing these environmental and knowledge externalities, demonstrably yield superior trajectories, fostering significantly higher and more effectively directed green R&D investment, leading to accelerated innovation, faster diffusion, substantially lower pollution, and maximized global welfare.

Furthermore, the model and simulations underscore the paramount importance of key parameters: the relative productivity of green versus brown R&D and the average intensity of green knowledge spillovers emerge as dominant drivers of the green transition’s pace and ultimate success. The elasticity of substitution between green and brown inputs also plays a significant role, particularly in determining long-run pollution levels. Finally, policy simulations reveal that while unilateral interventions provide benefits, globally coordinated policy mixes—specifically combining carbon pricing with green R&D subsidies—are markedly more effective, capable of approaching the optimal outcomes achieved under full international cooperation by synergistically addressing market failures related to both pollution externalities and innovation spillovers.

### Theoretical contributions

The principal theoretical advancement of this research resides in its novel synthesis of three core analytical frameworks—differential game theory, endogenous growth with directed technical change (DTC), and network analysis—into a unified dynamic international model. This integration uniquely allows the model to elucidate how strategic R&D competition, an element of differential game theory, endogenously determines the direction of technical change between "green" and "brown" technologies, a key feature of DTC models. Furthermore, it reveals how the network structure of international spillovers, a contribution from network analysis, critically mediates both the positive diffusion of innovations and the negative strategic incentives, such as free-riding, within a dynamic game context. The synthesized framework also captures the intricate feedback loops between national R&D strategies, the evolving global technological frontiers in green and brown sectors, and the consequent environmental quality.

Beyond these specific interactions, a significant contribution lies in demonstrating the multifaceted definition of "optimal policy"; policy choices that are optimal from a single country’s non-cooperative perspective will inherently diverge from those optimal for a coalition or for the global collective. Consequently, the explicit consideration of the networked nature of spillovers implies that even a country’s self-interested optimal strategy must account for the specific technological states and probable reactions of its network partners, thereby moving beyond analyses reliant on aggregate assumptions.

### Policy recommendations

The findings of this research underscore the exigent need for robust international cooperation that transcends conventional emissions targets to strategically coordinate green R&D endeavors and manage the intricate pathways of technology diffusion. Such concerted global action is paramount to surmounting the inherent inefficiencies and suboptimal outcomes arising from purely nationalistic policy approaches.

*Implement an Optimal Policy Mix*: A multifaceted policy approach is demonstrably superior to reliance on singular instruments. The insights from this paper strongly advocate for a combination of carbon pricing—to diminish the economic attractiveness of polluting "brown" technologies and internalize environmental externalities—and direct green R&D subsidies. The latter are crucial to correct for knowledge spillovers inherent in the innovation process and to proactively steer the direction of technical change towards environmentally sound solutions. This dual strategy effectively addresses both demand-pull and supply-push drivers of technological transformation, aligning with findings that suggest such mixes can closely approximate the optimal welfare outcomes of full international cooperation, thereby providing a powerful and politically feasible pathway to address climate externalities.

*Adopt Network-Aware Policy Interventions*: International and national policies must be cognizant of, and strategically leverage, the existing architecture of international R&D spillover networks. Efforts should be directed towards strengthening green spillover channels, which could involve fostering international research collaborations, supporting open-access knowledge platforms, and facilitating technology partnerships. Crucially, given the significant heterogeneity in national capabilities, enhancing the absorptive capacities of recipient nations, particularly developing countries, is vital to ensure that diffused green technologies are effectively adopted, adapted, and utilized. This is not a secondary concern but a prerequisite for the success of any global policy. It requires targeted supporting measures, such as large-scale investments in education, technical training, and institutional strengthening, financed through dedicated international transfer payments and technical assistance programs. Without these complementary policies, even well-designed global tax and subsidy schemes risk failure due to implementation gaps in nations with fiscal and absorptive capacity shortcomings.

*Actively Manage Brown Technology Spillovers*: A comprehensive green transition strategy must also address the persistent challenge posed by spillovers in brown technologies. The continued improvement and diffusion of emission-intensive technologies can significantly undermine or even negate progress made in green innovation. Consequently, policy frameworks should consider measures aimed at discouraging R&D in, and limiting the international transfer of, highly polluting technologies. This may involve coordinated international efforts to phase out support for such technologies and to ensure that development pathways globally are oriented towards sustainable alternatives.

*Establish Mechanisms to Mitigate R&D Free-Riding and Address Spillover Concerns*: The pervasive incentive for nations to free-ride on the R&D investments of others significantly curtails global innovation in green technologies. International agreements must therefore incorporate robust mechanisms to counteract this tendency, as simply calling for cooperation is insufficient; agreements must be designed to be incentive-compatible. Potential avenues include structuring conditional R&D commitments within frameworks like the G20, where national contributions are contingent on reciprocal efforts, or establishing an International Green R&D Fund to pool resources and distribute the costs and benefits of innovation more equitably. To directly address sovereign concerns over core technology spillovers, these agreements could incorporate a "Spillover Compensation Fund," financed by technology recipients, to reward innovator countries, thereby transforming the positive externality into a market-like transaction and making cooperation more politically palatable.

### Limitations and future research

This research, while comprehensive, carries certain limitations that open promising avenues for future inquiry.

First, the international spillover network is assumed to be exogenous and fixed. This is a primary limitation, as in reality, the ties that facilitate knowledge diffusion—such as R&D alliances, trade relationships, and foreign direct investment—are the result of strategic choices. Future work could endogenize the network structure itself. For instance, network links could evolve based on strategic decisions, where countries invest in forming R&D partnerships, or emerge from trade patterns that are themselves shaped by evolving comparative advantages in green or brown technologies. Such an extension would allow for the analysis of how policy and R&D competition not only operate on the network but also shape the very architecture of global innovation diffusion, potentially leading to the formation of "green tech clubs" or path-dependent fragmentation of the global knowledge system.

Second, the empirical grounding of our spillover network, while based on real-world trade data, has two key limitations. Firstly, our four-bloc network is a simplification. Future research should construct larger and more granular networks, for instance, by including all G20 or OECD countries. This would not only enhance the practical relevance of the policy simulations but also allow for a richer analysis of network metrics, such as community structures and the role of middle-man economies. Secondly, our reliance solely on trade data provides a partial view of spillover channels. While trade is a widely accepted proxy, a more comprehensive representation could be achieved by creating a composite index that incorporates other data sources. Future work could integrate data on international patent citations, foreign direct investment (FDI) flows, and scientific collaborations to build a multi-layered spillover network. This would provide a more robust and nuanced understanding of the complex pathways of global knowledge diffusion.

Third, the model’s results are contingent upon the specific functional forms chosen for production (CES), utility (logarithmic), and innovation. While these are standard in the literature, alternative specifications could alter the quantitative and potentially qualitative nature of the equilibrium dynamics and policy effectiveness.

Furthermore, our numerical analysis, based on an empirically-proxied network of major economic blocs, does not explicitly cover the unique circumstances of small open economies or resource-dependent countries. These nations often have limited capacity for frontier R&D and may be more vulnerable to brown technology lock-in due to entrenched industrial structures. The generalizability of our conclusions to these contexts requires further study, as the strategic incentives and policy constraints they face could differ significantly.

Finally, our analysis is purely economic and abstracts from political economy constraints. The implementation of "optimal" policies like global carbon taxes or the formation of cooperative agreements faces significant real-world hurdles, including industrial lobbying, national sovereignty concerns, and public acceptance, which are important areas for future integrated research.

## Figures and Tables

**Fig. 8:**
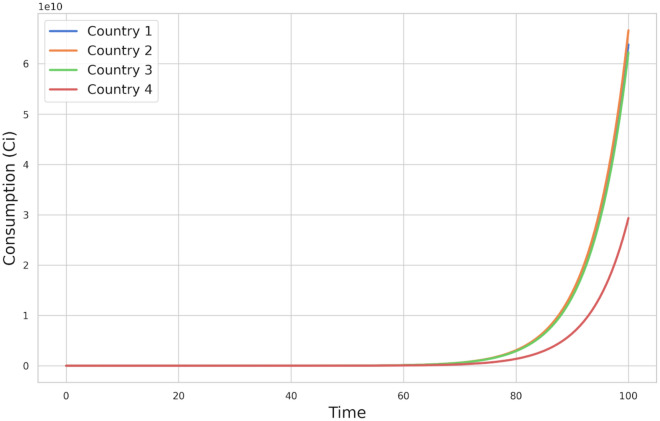
Individual country consumption paths

## Data Availability

No datasets were generated or analysed during the current study.

## Appendix A

Appendix Fig. 7 illustrates the individual technology stock trajectories for each country under the Nash equilibrium. The top panel shows the accumulation of green technology stocks, revealing relatively homogenous paths across the four blocs that align with the slow aggregate trend seen in Fig. 3. The bottom panel displays the paths for brown technology stocks; here, some divergence among countries is evident, with Country 2 and Country 3 showing the most rapid accumulation, reflecting the complex interplay of their R&D strategies and positions within the spillover network.

Appendix Fig. 8, illustrating individual country consumption paths, shows that consumption grows over time for all countries, broadly mirroring the trajectory of technological development. Differences in consumption paths appear relatively minor, suggesting the calibrated parameters do not induce strong asymmetries in this particular dimension in the baseline Nash outcome.

Appendix Fig. 9 provides a detailed breakdown of the individual R&D strategies for each of the four countries under the Nash equilibrium. The figure is organized into four rows, one for each country. For each country, the left panel depicts the path of total R&D investment ($I{R}_{i}$), while the right panel shows the corresponding share of that investment allocated to green R&D (${\theta }_{i}$). The total R&D investment paths (left panels) are qualitatively similar to the aggregate trend, each displaying an inverted U-shape with slight variations in peak levels and timing. The green R&D share paths (right panels) all follow the gradual upward trend observed in the aggregate Nash scenario, starting from relatively low levels and increasing modestly over the simulation period.

Furthermore, Appendix Fig. 10 highlights the evolution of the global brown technology stock under different scenarios. It vividly contrasts the unabated growth of brown technology in the Nash equilibrium with the outcomes under the cooperative solution and an illustrative "Policy Mix A". The cooperative solution achieves a significant suppression of brown technology accumulation, keeping its stock at a very low level. The "Policy Mix A" also demonstrates a considerable reduction in brown technology compared to the Nash outcome, though not as pronounced as under full cooperation, underscoring the effectiveness of policy interventions in steering technological development away from polluting pathways.

Collectively, the Appendix figures illustrate the cascading consequences of non-cooperative behavior. The suboptimal individual R&D investment levels and allocation shares shown in Fig. 9 directly result in the slow accumulation of green technology and the persistent growth of brown technology stocks at the national level (Fig. 7). While these technological trajectories support modest consumption growth (Fig. 8), they culminate in a significant global failure, as evidenced by the unabated expansion of the aggregate brown technology stock under the Nash equilibrium (Fig. 10). This underscores that without cooperative action or significant policy intervention, the strategic interactions of individual nations create a path-dependent lock-in, preventing the global economy from transitioning away from polluting technologies.

## Appendix B

The derivatives $\frac{\partial {Y}_{i}}{\partial {A}_{gi}}$ and $\frac{\partial {Y}_{bi}}{\partial {A}_{gi}}$ depend on the specific production functions (3)-(5) and labor allocation. For example, $\frac{\partial {Y}_{i}}{\partial {Y}_{qi}}\frac{\partial {Y}_{gi}}{\partial {A}_{qi}}={\alpha }_{Y}(\widetilde{{Y}_{i}}/{Y}_{gi}{)}^{1/\epsilon }{L}_{gi}$.The complexity of the spillover terms in the co-state equations highlights the network effects. Transversality conditions:

$$\underset{t\to \infty }{lim} {e}^{-{\rho }_{i}t}{\lambda }_{Pi}(t)P(t)=0$$

$$\underset{t\to \infty }{lim} {e}^{-{\rho }_{i}t}{\mu }_{gi}(t){A}_{gi}(t)=0$$

24 $$\underset{t\to \infty }{lim} {e}^{-{\rho }_{i}t}{\mu }_{bi}\left(t\right){A}_{bi}\left(t\right)=0$$

The OLNE is found by solving the system of $3N$ state equations (for ${A}_{gi},{A}_{bi},P$) and $3N$ co-state equations (for ${\lambda }_{Pi},{\mu }_{gi},{\mu }_{bi}$ for each country, although ${\lambda }_{Pi}$ might be common if $\phi$ and ${\rho }_{i}$ are symmetric), along with $2N$ algebraic equations from FOCs (15) and (17), subject to initial conditions and transversality conditions.

In an MPNE, strategies are functions of the current state vector $\mathbf{S}(t)=({\mathbf{A}}_{\mathbf{g}}(t),{\mathbf{A}}_{\mathbf{b}}(t),P(t))$.Each country $i$ solves:

25 $$\begin{aligned} {\rho }_{i}{V}_{i}\left(\mathbf{S}\right) &= \underset{{I}_{Ri},{\theta }_{i}}{max} \Bigg\{\mathrm{ln}\left({C}_{i}\right)-\frac{\phi }{2}{P}^{2}-\frac{{\chi }_{i}}{2}{I}_{Ri}^{2}\\ & \quad +\frac{\partial {V}_{i}}{\partial P}\dot{P}+\sum_{j=1}^{N} \left(\frac{\partial {V}_{i}}{\partial {A}_{gj}}{\dot{A}}_{gj}+\frac{\partial {V}_{i}}{\partial {A}_{bj}}{\dot{A}}_{bj}\right)\Bigg\} \end{aligned}$$

This is a system of N coupled Hamilton–Jacobi-Bellman (HJB) partial differential equations. The FOCs for ${I}_{Ri}$ and ${\theta }_{i}$ are similar in structure to (15) and (17), but with the co-state variables ${\mu }_{gi},{\mu }_{bi}$ replaced by the partial derivatives of the value function $\frac{\partial {V}_{i}}{\partial {A}_{gj}}$ and $\frac{\partial {V}_{i}}{\partial {A}_{bj}}$ respectively, and ${\lambda }_{Pi}$ by $\frac{\partial {V}_{i}}{\partial P}$.

For instance, the condition for optimal ${\theta }_{i}$ becomes:

26 $$\frac{\partial {V}_{i}}{\partial {A}_{gi}}{\eta }_{gi}{A}_{gi}^{{\nu }_{2}}=\frac{\partial {V}_{i}}{\partial {A}_{bi}}{\eta }_{bi}{A}_{bi}^{{\nu }_{2}}$$

Solving this system of HJB equations analytically is generally intractable for models of this complexity.

*Steady States*: A steady state (${{\mathbf{A}}_{\mathbf{g}}}^{*},{{\mathbf{A}}_{\mathbf{b}}}^{*},{P}^{*}$) would satisfy ${\dot{A}}_{gi}=0,{\dot{A}}_{bi}=0,\dot{P}=0$ and ${\dot{\mu }}_{gi}=0,{\dot{\mu }}_{bi}=0$, ${\dot{\lambda }}_{Pi}=0$ (for OLNE) or constant value functions (for MPNE).

*Comparison*: MPNE strategies are subgame perfect and time-consistent, unlike OLNEs. The nature of spillovers can critically affect R&D investment levels in MPNE versus OLNE. If strong positive spillovers in green tech exist, and country $i$ anticipates that its increased ${A}_{gi}$ will induce country $j$ to also increase ${A}_{gj}$ (which then spills back positively to $i$), this could amplify R&D incentives in MPNE. Conversely, if country $j$ is expected to free-ride by reducing its R&D in response to $i$’s advancements, $i$’s incentive to invest under MPNE might be lower than its pre-committed OLNE path. The specific structure of ${\Omega }_{g}$ and ${\Omega }_{b}$ and the strategic substitutability/complementarity of R&D efforts will determine these outcomes.

## Appendix C

### C.1 Introduction

This appendix provides a detailed account of the data sources, methodology, and resulting parameters used to construct the international spillover network matrices for both green technologies (${\Omega }_{\mathrm{g}}$​) and brown technologies (${\Omega }_{\mathrm{b}}$). Our objective is to anchor the simulation’s network structure in empirically relevant data, thereby ensuring transparency and enhancing the practical applicability of our findings. The methodology follows standard practices in the literature on international technology diffusion, where bilateral trade flows are used as a primary proxy for knowledge spillover channels.

### C.2 Green spillover network (${{\boldsymbol{\Omega}}}_{\mathbf{g}}$) calibration

#### C.2.1 Data source and rationale

For the green spillover network, we assume that knowledge diffusion occurs through general economic exchange and interconnectedness. Consequently, we utilize aggregate bilateral trade data as a comprehensive proxy for the pathways through which new ideas, processes, and technologies are transmitted. The data on total bilateral merchandise trade for the full year 2022 are sourced from the.

International Monetary Fund’s (IMF) Direction of Trade Statistics (DOTS) database. The analysis focuses on four major economic blocs that are central to global R&D and production: the United States (USA), the European Union (EU), China (CHN), and Japan (JPN).

#### C.2.2 Methodology

The spillover coefficient from a source country/bloc $j$ to a recipient country/bloc $i$, denoted as ${\omega }_{g,ji}$, is calculated based on import shares. This formulation captures the idea that the intensity of knowledge flowing from $j$ to $i$ is proportional to the importance of $j$ as a supplier to $i$. The specific formula is:

27 $${\omega }_{g,ji}={\kappa }_{g}\left(\frac{{\mathrm{Imports}}_{i\leftarrow j}}{\sum_{k\ne i} {\mathrm{Imports}}_{i\leftarrow k}}\right)$$

where:

- ${\mathrm{Imports}}_{i\leftarrow j}$ is the value of total merchandise imports by bloc $i$ from bloc $j$ in 2022.
- The denominator, $\sum_{k\ne i} {\mathrm{Imports}}_{i\leftarrow k}$, represents the total imports of bloc $i$ from all other blocs in the model.
- ${\kappa }_{g}$ is a scaling factor used to adjust the overall intensity of spillovers. It is calibrated to ensure that the average non-diagonal spillover coefficient in the network aligns with the baseline value of 0.05 specified in Table 2 of the main text.

Since ${\mathrm{Imports}}_{i\leftarrow j}$ is generally not equal to ${Imports}_{j\leftarrow i}$, the resulting spillover matrix ${\Omega }_{g}$ is asymmetric (${\omega }_{g,ji}\ne {\omega }_{g,ij}$). Therefore, the network is modeled as a directed and weighted network, where links have both a specific direction and an intensity. All subsequent discussions of network properties, such as centrality, will be based on this directed structure. This same principle applies to the brown spillover network ${\Omega }_{b}$.

The diagonal elements of the matrix, ${\omega }_{g,ii}$, are set to zero, as this matrix models international spillovers exclusively. Domestic knowledge dynamics are captured separately in the knowledge accumulation Eqs. (10) and (12).

### C.3 Brown spillover network (${{\boldsymbol{\Omega}}}_{\mathbf{b}}$) calibration

#### C.3.1 Data source and rationale

For the brown technology spillover network, we hypothesize that knowledge diffusion is more concentrated in mature, carbon-intensive sectors. Therefore, instead of using aggregate trade data, we focus on trade in specific goods that embody these technologies. We source our data from the UN Comtrade database for the year 2022, using the Standard International Trade Classification (SITC Rev. 4). We define a "brown goods" basket to include:

- Fossil Fuels: SITC 3 (Mineral fuels, lubricants and related materials).
- Heavy Industrial Machinery: A subset of SITC 7 (Machinery and transport equipment) known to be associated with traditional, energy-intensive manufacturing processes (e.g., machinery specialized for particular industries, power-generating machinery).

#### C.3.2 Methodology

The calculation methodology for ${\omega }_{b,ji}$ is parallel to that for the green network, but uses the trade data for our defined "brown goods" basket:

28 $${\omega }_{b,ji}={\kappa }_{b}\left(\frac{{\mathrm{BrownImports}}_{i\leftarrow j}}{\sum_{k\ne i} {\mathrm{BrownImports}}_{i\leftarrow k}}\right)$$

where:

- ${\mathrm{BrownImports}}_{i\leftarrow j}$ is the value of imports by bloc $i$ from bloc $j$ of goods within the specified "brown goods" basket.
- ${\kappa }_{b}$ is the corresponding scaling factor. In line with the assumption in Table 2 that emerging green technologies feature more potent knowledge spillovers than mature brown technologies, ${\kappa }_{b}$ is calibrated to achieve a lower average non-diagonal spillover coefficient of 0.02.

### C.4 Network properties and calibrated matrices

The application of the above methodologies to the 2022 trade data yields the following spillover matrices and descriptive statistics for our 4-node network. Table 4 presents the key descriptive statistics for the calibrated networks.

**Table 4:** Descriptive statistics of calibrated networks

Property	Green network (${\Omega }_{\mathrm{g}}$)	Brown network (${\Omega }_{\mathrm{b}}$)
Number of nodes (N)	4	4
Network density	1	1
Avg. link weight (non-diagonal)	0.05	0.02
Max. link weight	0.098 (EU to USA)	0.035 (CHN to USA)
Min. link weight	0.015 (JPN to CHN)	0.009 (JPN to EU)

The following matrices are used in the baseline numerical simulations. In each matrix, rows represent the recipient country ($i$) and columns represent the source country ($j$)

Green Spillover Matrix (${\Omega }_{\mathrm{g}}$)

$${\Omega }_{\mathrm{g}}=\left(\begin{array}{ccccc}& \mathrm{USA}& \mathrm{EU}& \mathrm{CHN}& \mathrm{JPN}\\ \mathrm{USA}& 0& 0.098& 0.075& 0.031\\ \mathrm{EU}& 0.095& 0& 0.065& 0.025\\ \mathrm{CHN}& 0.045& 0.055& 0& 0.015\\ \mathrm{JPN}& 0.035& 0.046& 0.020& 0\end{array}\right)$$

Brown Spillover Matrix (${\Omega }_{\mathrm{b}}$)

$$\begin{array}{c}{\Omega }_{\mathrm{b}}=\left(\begin{array}{ccccc}& \mathrm{USA}& \mathrm{EU}& \mathrm{CHN}& \mathrm{JPN}\\ \mathrm{USA}& 0& 0.028& 0.035& 0.012\\ \mathrm{EU}& 0.025& 0& 0.021& 0.009\\ \mathrm{CHN}& 0.018& 0.015& 0& 0.011\\ \mathrm{JPN}& 0.019& 0.016& 0.010& 0\end{array}\right)\end{array}$$
